# Continuous Monitoring of Health and Mobility Indicators in Patients with Cardiovascular Disease: A Review of Recent Technologies

**DOI:** 10.3390/s23125752

**Published:** 2023-06-20

**Authors:** Muhammad Ali Shiwani, Timothy J. A. Chico, Fabio Ciravegna, Lyudmila Mihaylova

**Affiliations:** 1 Department of Automatic Control and Systems Engineering, The University of Sheffield, Sheffield S1 3JD, UK; 2 Department of Infection, Immunity and Cardiovascular Disease, The Medical School, The University of Sheffield, Sheffield S10 2RX, UK; 3 Dipartimento di Informatica, Università di Torino, 10124 Turin, Italy

**Keywords:** prognosis and health management, patient monitoring, activity recognition, biomedical monitoring, indoor localisation, wearable devices, electrocardiography, photoplethysmography, cardiovascular disease, remote monitoring

## Abstract

Cardiovascular diseases kill 18 million people each year. Currently, a patient’s health is assessed only during clinical visits, which are often infrequent and provide little information on the person’s health during daily life. Advances in mobile health technologies have allowed for the continuous monitoring of indicators of health and mobility during daily life by wearable and other devices. The ability to obtain such longitudinal, clinically relevant measurements could enhance the prevention, detection and treatment of cardiovascular diseases. This review discusses the advantages and disadvantages of various methods for monitoring patients with cardiovascular disease during daily life using wearable devices. We specifically discuss three distinct monitoring domains: physical activity monitoring, indoor home monitoring and physiological parameter monitoring.

## 1. Introduction

The World Health Organisation estimates that the global population of people over 60 years old will double from 12% in 2015 to 22% by 2050 [1]. Ageing increases the risk of many diseases, including diabetes, heart diseases, osteoarthritis and dementia.

Cardiovascular diseases (CVDs) are the leading cause of death globally [2]. CVDs cause various manifestations ranging from no symptoms to heart failure, stroke and sudden death. There are several risk factors for CVDs that can be influenced to an extent by a person’s behaviour: physical inactivity, high blood pressure, abnormal blood lipids, obesity and smoking. Four of these (excluding smoking) can directly be linked to physical inactivity, as regular exercise reduces blood pressure, reduce LDL cholesterol levels and promotes weight loss [3].

**Figure 1:**
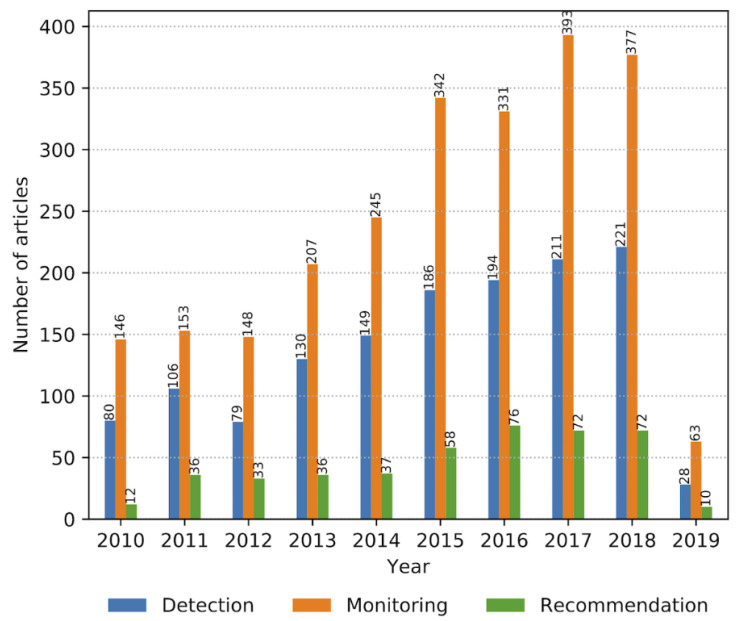
Number of publications relating to health monitoring wearables that provide a user with information in the form of recommendations, monitoring and detection. From January 2010 to February 2019. Reprinted with permission from [6].

CVDs, ageing, physical activity and mobility are intertwined in a complex manner and are influenced by an individual’s behaviour and physical condition. For example, a review of studies relating to frailty in patients with CVD by Afilalo et al. [4] concluded that frailty increases the risk of CVDs, and that CVDs can cause frailty, with them in combination increasing mortality. Monitoring these factors provides insights that could assist clinicians in preventing, diagnosing and managing CVDs. In addition, there is a need for better remote monitoring to reduce healthcare costs by obtaining data outside clinical settings [5]. This has been magnified by the COVID-19 pandemic, in which access to clinical settings has been greatly restricted. Advances in mobile health technologies have allowed for the continuous monitoring of key health and mobility indicators during free living. Figure 1 shows the number of articles each year relating to health monitoring based on wearable systems that provide a user with information in the form of recommendations, monitoring and detection.

This review discusses these new and emerging technologies and how they measure indicators of cardiovascular health. Our focus is on existing Internet-of-Things (IoT) technologies that can be used in daily life relatively easily, particularly constant-wear devices for continuous monitoring. A range of these exist, including wrist-worn devices, glasses, smart textiles and rings. However, many are still within the early stages of adoption, and have limitations such as washing (textiles), lack of utility/relevance (e.g., smart glasses) and high cost. Wrist-worn wearables are the most widely adopted [7]. This enables a set of constraints to be developed from a clinical perspective based on existing technologies and highlights areas for further research.

Although literature reviews are available on the three areas covered in our paper, most of these focus on a single domain. Works in [8,9] discuss wearable technology for monitoring general health and wellbeing for the ageing population. The work in [10] is on cardiac health, but focuses on data analysis rather than data acquisition. Ref. [11] discusses both data acquisition and analysis in the cardiac domain, but detail on physiological parameters is limited, and physical activity and indoor localisation are not discussed. Similarly, various reviews consider only on the technological side of indoor localisation, and only briefly mention health monitoring as a potential application, such as [12,13]. Wang et al. [14] review all three aspects, but focus on elderly care, and the monitoring domains are not discussed in terms of cardiac-specific health apart from a brief mention of some CVDs in the physiological parameter monitoring section.

This paper discusses three distinct monitoring domains. Section 2 describes technologies that can monitor the general physical activity of a user. Section 3 presents technologies that assess indoor home activity. Section 4 reviews the sensors that are available on constant-wear devices and how they monitor physiological parameters, as well as the significance of these as cardiovascular health indicators.

## 2. Physical Activity Monitoring

### 2.1. Step Counting

A step counter is the basic activity monitoring function of all wearable devices. Total daily steps can be displayed to the user on the wearable itself and/or on an accompanying application, often alongside a set target, such as 10,000 steps [15]. The number of daily recommended steps is 6500–7500, according to Ayabe et al. [16], for the secondary prevention of CVD based on the correlation with the total physical activity energy expenditure. Additionally, measuring steps over time (cadence) allows for the measurement of activity intensity. Along with monitoring a patient’s activity levels, a step counter has been proven to encourage patients to increase physical activity, aiding in the prevention of obesity, CVDs and rehabilitation [17,18,19]. The optimal target steps/day remains uncertain; but increasing physical activity from any baseline provides health benefits.

Steps are measured using a three-axis accelerometer that converts raw acceleration measurements into steps. The algorithms used to derive steps from accelerometer data vary between each device and must consider device placement; for example, hip-worn devices produce different acceleration signals compared to wrist-worn devices [20].

The accuracy of step counters is variable, and may not always be the best measurement to infer physical activity. In particular, inaccuracies are related to slower walking speeds, which are more prevalent in older, frail patients [21]. Additionally, Pepera et al. [22] concluded that patients with chronic heart failure have a lower cadence and a shorter step length than healthy counterparts. Thorup et al. [23] found that a walking speed of at least 3.6 km/h is required for accurate step count measurement using a Fitbit Zip activity step tracker. Inaccurate measurements can also arise from excessive arm movement with wrist-based step counters, resulting in inaccurate “step” counts.

A validation study for consumer-level activity monitors by Vetrovsky et al. [24] is summarised in Table 1. This shows that heart failure patients had lower step counts measured by all devices, as well as a higher mean absolute percentage error (MAPE). The decrease in MAPE from the Garmin Vivofit 1 to the Garmin Vivofit 3 suggests an improved step count measurement in the newer device. The exact algorithm used to calculate steps from accelerometer data is usually proprietary to the company, and it is difficult to quantify improvements in newer generations without further validation. These are challenging due to the rapid rate of new iterations outpacing the time required for such validation [25].

### 2.2. Raw Accelerometer Data

A study by Ramezani et al. [26] on subacute rehabilitation patients with limited mobility used raw accelerometer data to measure physical activity. The Sony SmartWatch 3 was used to collect data at 16 Hz used in (1) to calculate the signal magnitude, where $F_{x}$, $F_{y}$ and $F_{z}$ are the forces around each axis on the three-axis accelerometer.

(1) $$Signal\;Magnitude=\sqrt{F_{x}^{2}+F_{y}^{2}+F_{z}^{2}}.$$

The signal magnitude obtained is passed through a fifth-order Butterworth band-pass filter in batches of 10 s with cut-off frequencies of 0.5 and 8 Hz, which limits the signal to frequencies common in human motion. It calculates the mean absolute deviation of the signal magnitude, which represents the average magnitude of acceleration within the 10 s interval and is directly proportional to the force applied to the watch through the equation f = ma. This is used to calculate energy to quantify patient activity. It uses an algorithm to determine whether a patient is active, inactive or stationary active.

A framework by Kheirkhahan et al. [27] for real-time mobility monitoring uses a similar method to infer physical activity from accelerometer data instead of steps. The framework includes an application for the Samsung Gear S2/S3, which collects data at 10 Hz and processes them in batches of 15 s into (1) to obtain the signal magnitude. Cut-off frequencies for this system are 0.6–2.5 Hz. The computations are performed on the smartwatch itself, and only the variables are transmitted to a remote server by HTTPS communication. The calculation and data cleaning significantly reduce the amount of data stored on the watch and transmitted to the server via WiFi or cellular network. The server analyses the data to quantify daily minutes spent sedentary, and in light, moderate, vigorous and total activity.

**Figure 2:**
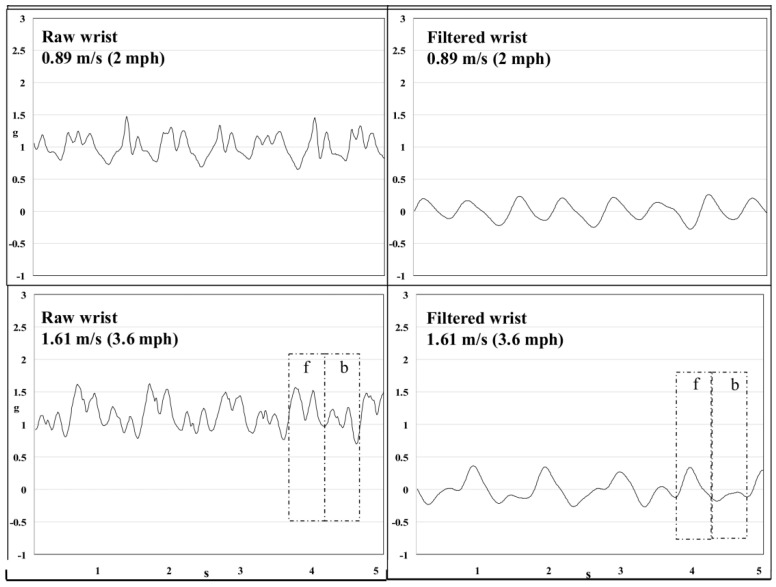
Signals measured on the y-axis of an accelerometer at 80 Hz in a 5 s window placed on the wrist of a single participant walking at different speeds. Filtered signal is obtained by applying a 4th-order bandpass filter with cut-off frequencies 0.25–2.5 Hz. ‘f’ denotes a forward arm swing. ‘b’ denotes a backward arm swing. Reprinted with permission from [20].

Using raw accelerometer data allows for advanced activity recognition, which can be accomplished using data mining and machine learning to develop activity models [28]. After sensor data collection, it is filtered (Figure 2) and segmented for feature extraction. The most significant features associated with the activity are used for classification. A survey by Mostafa et al. [29] describes the different features that can be extracted from accelerometer data, including mean, skewness, root mean square and power spectral density. The survey also provides a list of activities that have been detected, including activities of daily living (ADLs), such as kitchen use and toileting, which can be used to monitor mobility and frailty [30].

Sensor placement is key, because activities produce different signals based on the movement of a specific part of the body during activity. Algorithms such as that in [31] are exclusive to wrist placement, as it recognises hand gestures to determine whether a patient has taken a medication. Atallah et al. [32] conducted a study with 11 participants to determine the best position to place an accelerometer for different types of activities. Activities were placed into five groups: very low-level activity (lying down), low-level activity (socialising, getting dressed, reading, etc.), medium-level activity (vacuuming, walking 2 km/h, etc.), high-level activity (running 7 km/h, cycling, etc.) and transitional activities (sitting down and getting up, lying down and getting up). The study found that the optimal placement varied between the different activity groups, with wrist placement being optimal for very low-level activities. Using a combination of sensors provides better accuracy compared to a single sensor. Integrating location within the activity recognition algorithm further reduces errors as it provides the context of the activity, whereby a kitchen activity can be labelled as false if the user is located elsewhere. A study by Ceron et al. [33] that combined smartwatch/phone accelerometer data with location data to classify sedentary behaviour showed a significant improvement in precision compared to accelerometer data alone. Bluetooth Low Energy (BLE) beacons were placed at points of interest to obtain location data.

### 2.3. Gyroscope Data

As sensors able to measure angular velocity and the rate of rotation around an axis, gyroscopes have been increasingly incorporated into wearable devices for physical activity monitoring. These can provide insights into the user’s motion and orientation over time. Gyroscopes are often used in conjunction with accelerometers and can improve accuracy of activity recognition by capturing rotational movements not easily detected by accelerometers alone. This can enhance the performance of step counting algorithms, as both sensors capture different parts of the gait cycle [34]. Gyroscopes provide superior accuracy when used alone in certain activities, such as walking up or down the stairs, compared to accelerometers [35]. However, they consume significantly more power than accelerometers, which is a concern for wearable devices with limited battery life [36]. Furthermore, processing and analysing gyroscope data requires advanced algorithms and computational resources, which can be demanding for resource-constrained wearable devices.

Gyroscope-based activity recognition can provide information on the intensity and quality of daily movements, which can aid in managing and monitoring cardiovascular diseases. For instance, the study in [37] uses a gyroscope to identify sit-to-stand and stand-to-sit movements, which have shown to be reliable measures for patients undergoing cardiac rehabilitation for conditions including myocardial infarction, hypertension and coronary artery bypass grafting [38]. Additionally, Horsman et al. [39] validated the sit-to-stand movement as a noninvasive method for cardiac baroreflex assessment by measuring the changes in blood pressure during the movement. In the study, the movement was manually initiated, and the blood pressure was measured using a Finometer. However, with wearable devices, this measurement may be fully automated and taken throughout the day by detecting when a patient stands up using the gyroscope and measuring the changes in blood pressure using the sensors available, which are described in detail in Section 4.

### 2.4. Magnetometer Data

Magnetometers measure the magnetic field strength and orientation, providing information about a device’s position relative to Earth’s magnetic field. They are frequently used alongside accelerometers and gyroscopes, complementing the data to recognise physical activity and ADLs [40]. Studies using only magnetometers for activity recognition are limited due to their poor performance and power consumption compared to accelerometers and gyroscopes [36]. A study by Shoaib et al. [35] compared the three sensors, and suggests a supporting role for the magnetometer rather than a lead role in activity recognition. They suggest selecting features that are less sensitive to its direction dependence for better performance. Barnes et al. [41] used a wrist-worn magnetometer to assess fundamental movement skills in children by capturing the motion pattern for nine activities in a consecutive circuit, including overarm throw, sprinting, and balance bench. The data were clustered using multidimensional scaling to produce a ranking measure used to compare the relative abilities of the children in the cohort. A similar approach can be used to assess frailty and assist in clinical decision making, such as the treatment pathway for patients with aortic stenosis [42,43].

In any study or system, it is important to assess the monitoring requirements and the participants’ general mobility levels to inform whether to use the default step count or raw sensor data (provided this is accessible). An available validation study at a low step cadence for the device being used in the study would also assist in decision making. However, it is unlikely that a validation study will be available for the latest devices.

## 3. Indoor Home Tracking

Life-space can be defined as the extent, frequency and independence of an individual’s mobility [44]. This can be divided into patterns of areas that extend in distance from where an individual sleeps. A study by Peel et al. [45] on life-space assessment defined a model categorising areas into six life-space levels; bedroom, home, outside house, neighbourhood, town and unlimited. Life-space correlates with health outcomes, mobility and physical performance [44]. Measuring an individual’s life-space can provide valuable information on their daily routines, wellbeing and activities to help understand their behaviour and effect of CVDs. For example, a CVD patient experiencing fatigue may show a reduced life-space. On the other hand, a CVD patient on a good day may show reduced activity but with an extended life-space due to using a different mode of transport. Life-space outside the home can be measured using GPS, which is available in all smartphones and some smartwatches. GPS is considered to be the gold standard, but cannot be used indoors due to poor signal [46].

An average person spends a significant amount of time within their home, which consists of areas where they conduct daily activities such as sleeping, cooking, toileting and leisure time. Thus, indoor home tracking can help understand the daily life of a patient, based on the time they spend in different areas in their homes and their activities in them.

Indoor localisation can be implemented using a variety of different technologies and techniques [12,47]. Many of these have been developed for industrial settings, wireless sensor networks and robotics. Advances in wireless communication capabilities for mobile phones and wearable devices allow for the localisation of their users [12]. Indoor localisation requires the indoor environment to be known before a user can be mapped inside of it. Many studies develop systems based on a single, familiar environment such as a shopping centre [48], hospital [49] or rehabilitation facility [26]. Whilst the technologies used by these systems can be applied to a generic home, a different approach is required to map individual homes, which must be easy to perform by the user. Indoor localisation is typically achieved using short-range communication technologies that estimate relative indoor location with respect to reference points [12]. Indoor settings are complex, and contain obstacles such as walls and furniture, which affect signal propagation and reduce line-of-sight (LoS) propagation [50]. Transmitted signals can arrive at the receiver through different paths (multipath) due to effects such as reflection, refraction and diffraction [51]. Advanced systems may locate a user to a high degree of accuracy, but at minimum, a system is required to locate the room or particular area of interest that a user is in [52]. With any indoor tracking system that requires the user to carry a device, it is important to ensure that the device is suitable for long periods of use. Some studies use reference points with mobile phones [44,53,54]; however, this is not practical in a home setting where users do not carry their mobile phone at all times [9]. Wearable devices are more suitable for this.

### 3.1. Localisation Techniques

#### 3.1.1. Received Signal Strength Indicator (RSSI)

The received signal strength indicator (RSSI) is a widely used approach for indoor localisation [55,56]. It works by estimating the distance between a transmitter (*Tx*) and a receiver (*Rx*) based on the received signal strength (RSS) at the *Rx* using the path-loss propagation model described in (2), [57]:

(2) $$\mathrm{RSSI}=-10n\log_{10}\left(d\right)+A,$$

where *d* is the distance between the Rx and Tx; *n* is the path loss exponent; and A is RSSI value at a reference distance from the receiver [12]. The RSSI value decreases as the Tx moves further away from the Rx.

**Figure 3:**
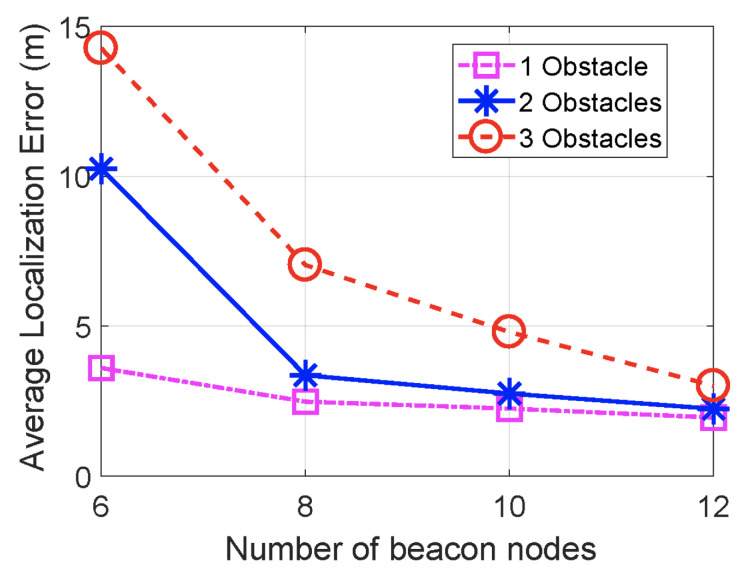
Results from study showing localisation accuracy increasing with increasing number of beacon nodes and decreasing with higher number of obstacles. Reprinted with permission from [59].

**Figure 4:**
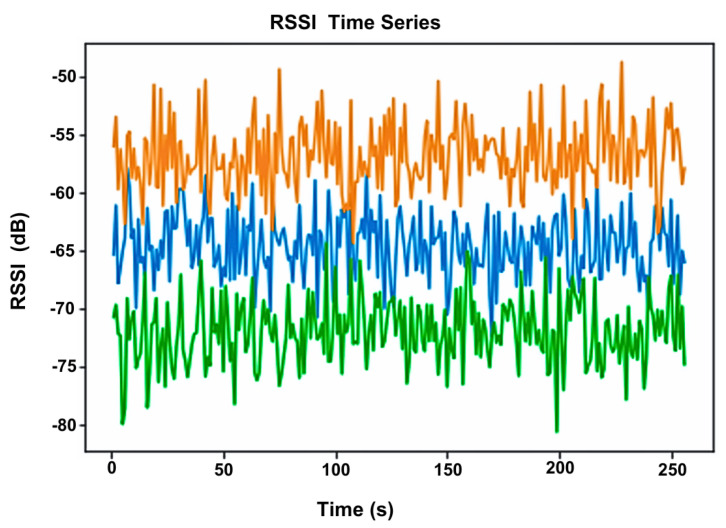
Variation in RSSI values from three access points using WiFi technology. Obtained from a person with a device standing in a fixed location. Each access point is at different distance from the user, with the orange, blue and green signals representing access points with increasing distances respectively. Reprinted with permission from [56].

**Figure 5:**
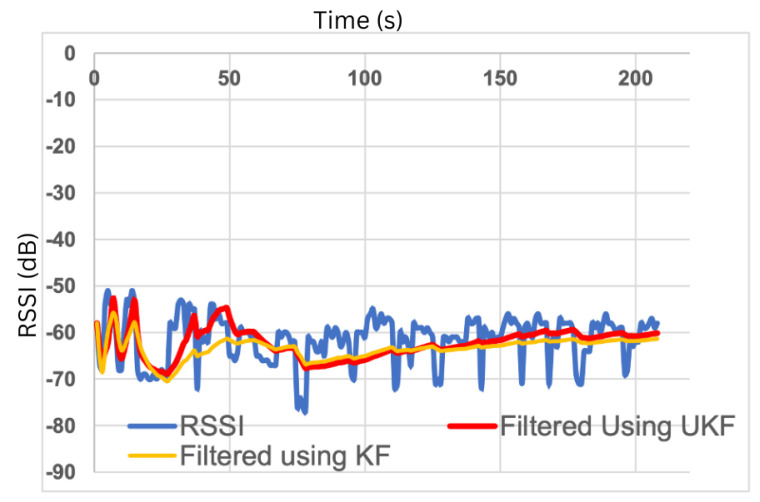
Application of the Kalman Filter (KF) and unscented Kalman Filter (UKF) to reduce noise on RSSI values. Reprinted with permission from [60].

The RSSI signal is low-cost and easy to collect. However, it is unstable and has poor accuracy, suffering from multipath effects of an indoor environment and non-line-of-sight (NLoS) paths. Figure 3 shows the effect of obstacles on the localisation error. Figure 4 shows the large fluctuations in the RSSI whilst devices are in the same position. Complex algorithms are required to mitigate these effects; the Kalman filter, used in [9,58] and displayed in Figure 5, is often used to smooth out RSSI values.

#### 3.1.2. Channel State Information (CSI)

Orthogonal frequency division multiplexing (OFDM) is widely used in wireless communication applications, including WLANs, where data are modulated on multiple subcarriers in different frequencies. Channel state information (CSI) captures the amplitude and phase responses of the channel in each subcarrier and between separate *Tx*-*Rx* antennae pairs. CSI can be described using (3):

(3) $$H\left(f\right)=\left|H\left(f\right)\right|e^{jsin(∠H)},$$

where H(f) is the CSI at the subcarrier with frequency *f*, ∠H is the phase and |H(f)| is the amplitude. CSI provides richer multipath information and factors in the combined effect of path-loss phenomena such as scattering, fading and power decay [12,61]. It is much more stable in indoor settings and has the potential to achieve submetre accuracy [62]. RSS in comparison only estimates the average amplitude of the entire signal bandwidth and the signal over all antennae [12]. A review by Alhomayani et al. [50] stated that there was no implementation of smartphone usage to collect CSI data at the time of its writing. This is due to operating systems not allowing access to the lower levels of the network stack, which is required to extract CSI data [63]. Accessing these data may be possible in the future, which would allow for the implementation of this technique with wearable devices. Current implementations utilise WiFi network interface cards (NICs) [64].

#### 3.1.3. Angle of Arrival (AoA)

The Angle of arrival (AoA) method uses an antenna array at the *Rx* (access point), which measures the phase at which the transmitted signal arrives at each antenna. The phase difference at each antenna is used to calculate the incident direction of the signal [65]. The time difference of arrival at each antenna is used to calculate the distance. AoA only requires three access points to estimate the location of a device in 3D space, and can be more accurate than RSSI in shorter distances, as the received signal phase is more stable than RSS. However, the accuracy decreases as the *Tx*-*Rx* distance increases. Antenna arrays are expensive, complex to build and have a higher power consumption than RSS [65,66].

#### 3.1.4. Time of Arrival (ToA)

Time of arrival (ToA) uses the velocity of the signal wave to calculate the distance between a Tx and Rx by measuring the time between when the signal was transmitted to when it was received [67]. For localisation, trilateration can be used with three reference points, similar to RSSI. However, if a line-of-sight path is not available, the signal may arrive via a longer path, which will increase the time and cause errors in the distance measurement. ToA also requires synchronisation between Tx and Rx and clocks with high resolutions, which adds to the cost and complexity of a system [12,67].

#### 3.1.5. Return Time of Flight (RToF)

Return time of flight (RToF) uses the same principle as ToA, but instead of measuring the time for a single transmission from Tx to Rx, it measures the round trip, Tx−Rx−Tx. The technique is also called two-way ToA [67]. It requires a less strict clock synchronisation than ToA. However, the calculations are more prone to errors due to the signal propagating twice.

#### 3.1.6. Time Difference of Arrival (TDoA)

Time difference of arrival (TDoA) exploits the difference in velocities for different signals that are transmitted at the same time or after a fixed time interval to calculate the distance based on the different times of arrival of each signal. Synchronisation is required only between the transmitters, and the technique does not require the transmitted signal time. However, more complex hardware is required to implement transmitting signals at different velocities, and TDoA suffers from the same lack of line-of-sight issues as ToA [67].

### 3.2. Localisation Methods

#### 3.2.1. Range-Based Method

**Figure 6:**
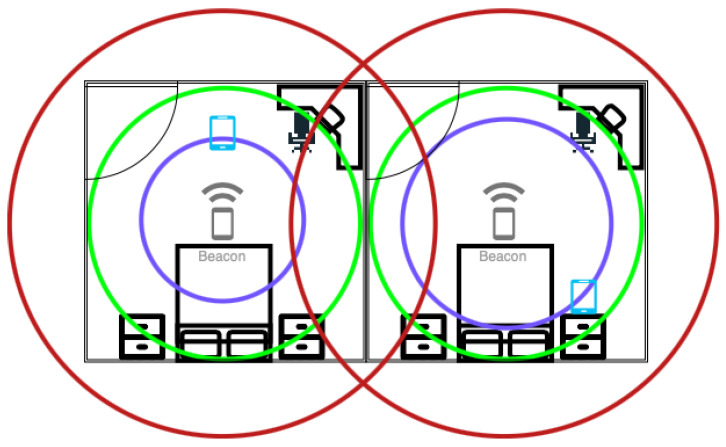
Example of geofencing through RSSI. The beacons in the middle of the rooms are the reference points. The red circle is the maximum radius of the beacon, green circle is the radius of the room (the geofence) and the purple circle is the radius where the devices have been detected.

The exact location of a device relative to reference points can be determined by using trilateration or multilateration [52]. This requires the distance between the device and at least three reference points in a 2D map. Geofencing can be used to determine the proximity of a device relative to a reference point. A geofence is a defined area around a reference point with distance thresholds; values within this threshold determine the device to be inside the geofence [68]. An example can be seen in Figure 6, where a reference point is placed in the centre of a room with a geofence approximately the size of the radius of the room, determining whether a device is inside or outside the room.

#### 3.2.2. Fingerprinting Method

**Figure 7:**
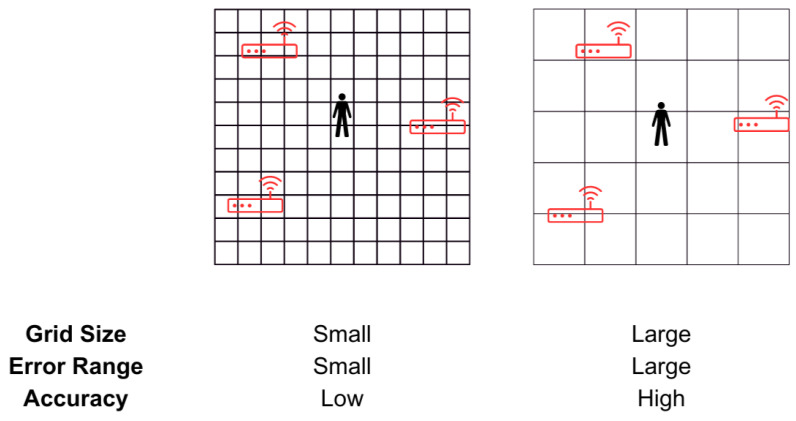
Example of grid-based fingerprinting, where the map is divided into a grid and the location is estimated to a specific cell.

Fingerprinting is a localisation method that can be implemented using the techniques described above. The process involves two steps: the training phase and the operating phase. The training phase consists of collecting measurements (RSSI, CSI, AoA, etc.) at predefined points to build a database of fingerprints, where each measurement corresponds to the defined location [56]. During the operating phase, the measurements obtained are compared against the trained fingerprint database to estimate the location of the user. Using this method allows for the mitigation, to an extent, of the issues caused by multipath signals, as the measured signals in the training phase are affected by the same environment [69]. However, changes in the environment, such as movement of furniture, may lead to estimation errors and require fingerprints to be repeated [56]. Higher location precision and accuracies can be achieved by collecting more fingerprints up to an extent, but this increases the time, cost and complexity of the training phase [12,56,70,71]. Machine learning algorithms such as artificial neural networks (ANN), k-nearest neighbour (kNN) and support vector machines (SVM) are used to match the online measurements with the fingerprints [12]. An illustration of grid-based fingerprinting is shown in Figure 7.

**Figure 8:**
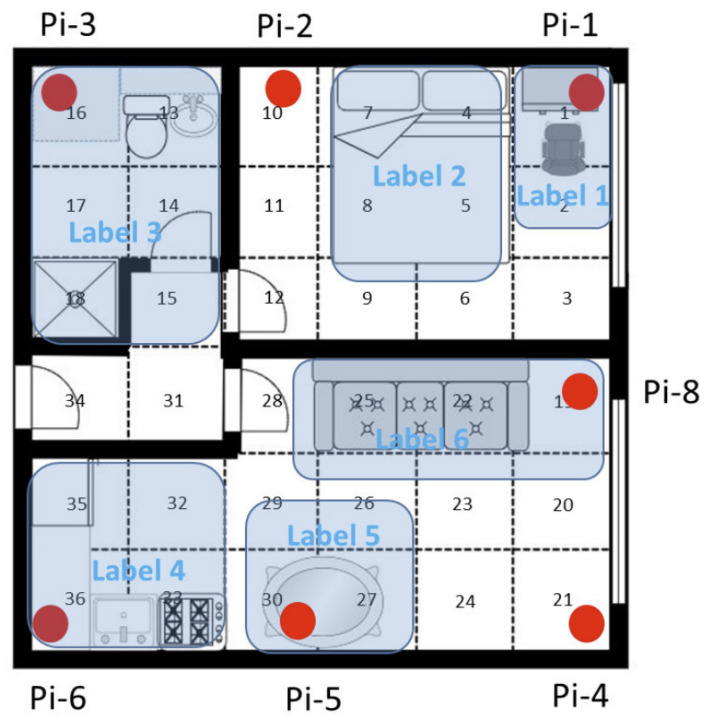
Location -of-interest-based fingerprinting using 6 areas of interest. Red circles denote the Rx. Reprinted with permission from [9].

In a generic home, it is not feasible to create maps to mark the reference points or create grids where fingerprints can be taken. Systems in [9,72] use location-of-interest-based fingerprinting in the training phase, where the user is required to be in close vicinity of a scanner to classify the RSSI fingerprints. Ref. [9] suggests a user can use a mobile app to annotate the different scanners placed around their homes, as illustrated in Figure 8.

### 3.3. Indoor Localisation Technologies

#### 3.3.1. Bluetooth Low Energy (BLE)

Bluetooth Low Energy (BLE) is the newest version of Bluetooth, which allows BLE beacons to transmit signals at periodic intervals. BLE devices can constantly scan for these signals, allowing for data transfer along with an RSS [12]. Although other techniques such as AoA and ToF can be used for localisation, RSSI is the most prominent one [73]. BLE is low-cost, has a low power consumption and is easily deployable [74]. Battery-powered beacons can be positioned towards the ceiling, reducing the shadowing effects caused by furniture.

BLE can be used in two strategies: the first is where scanning devices are placed at fixed locations with the user carrying a transmitting beacon; the second is where the user carries the scanning device, and the beacons are placed at fixed locations within an indoor setting [75]. The scanning device has a higher power consumption due to the constant scanning and processing of the received data, which needs to be taken into consideration when deciding on the orientation. For example, where it is not convenient for a user to have to charge a tracking device, they should not carry the scanning device.

#### 3.3.2. WiFi

WiFi (IEEE 802.11) localisation is a highly researched area due to the mass availability in consumer devices and existing infrastructure [58]. RSSI, CSI (exclusive to WiFi [76]), ToF and AoA can be used with WiFi signals to achieve device localisation using WiFi access points [12], with most studies using RSSI and fingerprinting [77]. The positioning of WiFi access points is dependent on the availability of mains sockets as they require constant power [58]. Power consumption on the user device is also higher compared to BLE localisation, and it requires complex processing algorithms, as WiFi was not originally designed for localisation [12].

#### 3.3.3. Radio Frequency Identification Device (RFID)

Active RFID is a low-cost, battery-powered technology that is capable of transmitting data hundreds of metres away from an RFID reader, making it ideal for localisation. However, it is not readily available on consumer devices [12]. Passive RFID, in comparison, is widely available, but only has a range of 1–2 m, making it unsuitable for passive indoor localisation [12].

#### 3.3.4. ZigBee

Zigbee builds on the IEEE 802.15.4 standard for low-rate wireless personal area networks that are low-cost and energy-efficient [78]. Solutions mainly use the RSSI technique, and localisation can be achieved via fingerprinting and range-based methods [79]. Zigbee is not readily available on the majority of user devices, and hence, it is less popular than WiFi and BLE in indoor localisation systems [12].

#### 3.3.5. Visible Light Communication (VLC)

**Figure 9:**
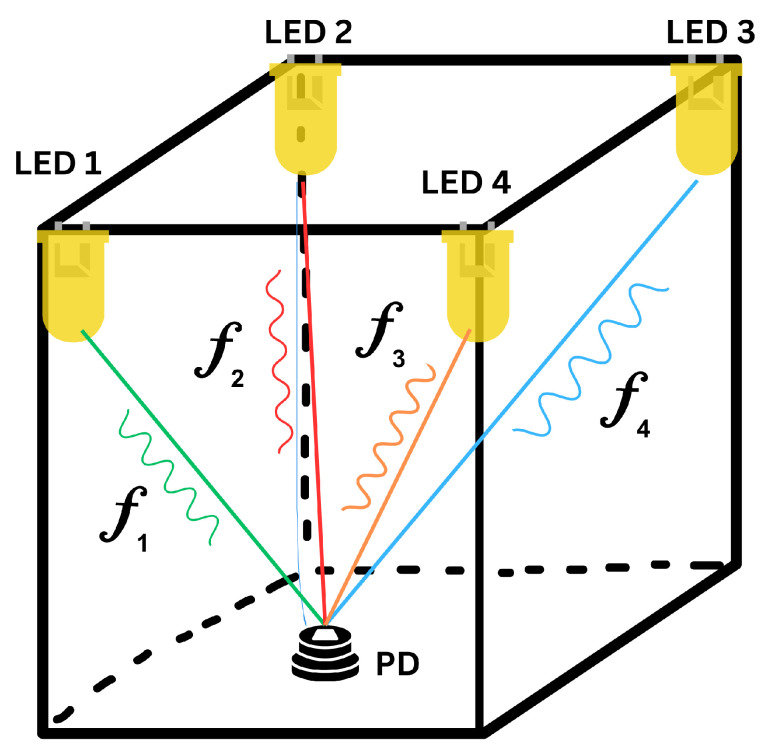
Illustration of indoor positioning using visible light using LEDs and a photodiode (PD).

Visible light communication (VLC) makes use of LEDs that emit signals to be received by photodiodes or cameras. Photodiode-based systems are compact, have a low power consumption and do not require complex processing. Camera-based systems are less susceptible to errors from external conditions such as ambient light, and generally perform better in nonideal environments [80]. The hardware is usually simple, low-cost and does not cause any electromagnetic or radio frequency interference. A high level of accuracy can be achieved using the AoA technique, whilst other techniques, including RSSI, TOA and TDOA, can also be used [81]. However, obstacles can block signals, and this is dependent on the line-of-sight path [12,82]. Figure 9 gives an illustration of VLC indoor positioning.

#### 3.3.6. Acoustic Signal

Acoustic signal localisation works by capturing sounds emitted by reference nodes in microphones that are commonly found on smartphones and some smartwatches. The ToA technique is used to calculate the distances between the speaker and the microphone using the speed of sound. A low transmit power is required in order to make the sound unnoticeable, which requires complex signal processing at the microphone end of the system. The microphone needs to be constantly listening for the acoustic signals, which reduces battery life [12,83].

#### 3.3.7. Ultrasound

Ultrasound works using the ToF technique, which uses the speed of sound to calculate distance between a Tx and Rx. Ultrasound can achieve a high level of accuracy compared to other technologies whilst being energy-efficient and low cost. However, it is dependent on a line-of-sight transmission and coherent sensor placement, which is difficult to obtain when tracking a human with complex movements [84].

#### 3.3.8. Ultrawideband (UWB)

Ultrawideband (UWB) makes use of the TDOA technique for short-range, high-bandwidth communication. UWB can achieve accuracies of up to 10 cm, and the signals are less prone to multipath effects. The UWB standard is slow to progress; hence, it does not feature in many consumer devices [12,14].

#### 3.3.9. The Fifth Generation of Mobile Communications (5G)

The fifth generation of mobile communications is based on OFDM, and has features including ultradense networks (UDN), millimetre waves (mm-wave) and massive multiple-input-multiple-output (MIMO), which enable high-accuracy indoor positioning [85]. UDN and massive MIMO, in particular, make 5G very promising for positioning, as they increase the likelihood of having a LoS signal [86]. The envisioned massive machine-type communications (mMTC) would increase the number of reference nodes that a user device can be localised through using device-to-device (D2D) communications [87]. These 5G devices can use RSSI, TDOA, AOA, RToF and CSI techniques. This 5G technology is still in the early stages of deployment, and is not widely available.

#### 3.3.10. Light Detection and Ranging (LiDAR)

Light Detection and Ranging (LiDAR) emits a near-infrared laser pulse at a high rate and uses the RToF technique to calculate the distance between the scanner and the target. The distance information can be used to create maps of an environment with a high level of accuracy [88]. LiDAR sensors have recently been integrated on high-end consumer devices such as the iPhone 12 Pro, and are likely to be a part of mainstream devices in the future [89]. Such devices are also equipped with cameras, enabling LiDAR and camera fusion, which allows for the 3D modelling of an indoor environment [90]. The camera images are higher-resolution and provide colour, whilst LiDAR provides accurate range measurements [91].

### 3.4. Requirements for Combining Different Methods

Most studies that focus on developing a system using a specific technology present arguments against other technologies to justify the development of their own system in a specific context (e.g., in [82,83]). The choice of technology and techniques depends on the application of a given system. A review by Wang et al. [14] provides a list of the significant performance metrics that need to be considered in the selection of a technology: (1) accuracy; (2) user privacy; (3) coverage area; (4) required user-side device; (5) cost; (6) complexity; (7) continuity; (8) update rate; (9) data output.

Using these performance metrics, the review also developed a summary of requirements that are applicable to a system developed for elderly care, shown in Table 2.

Some studies focus on innovative implementations that aim to improve on one or more performance metrics within the context of their own application. Three examples are briefly described below.

A study by Kolakowski et al. [92] developed a system that combines BLE and UWB technologies to provide room-level accuracy with BLE RSSI and submeter accuracy with UWB TDOA. The user-carried tag transmits UWB packets used for localisation only when the on-board accelerometer detects motion, to conserve power. BLE packets are transmitted every 5 s when stationary, and include data from other sensors.

Surian et al. [93] developed a novel system that uses BLE with a received number of signals indicator (RNSI) instead of the widely used RSSI. Transmitting beacons were placed around the environment, broadcasting a signal every 100 ms, with the user carrying the scanner. RNSI values decrease more consistently with increasing distance compared to RSSI values. A higher RNSI coming from a beacon maps the user in the location of that beacon. The system aims to avoid a complex fingerprinting phase using this method. An experiment for comparison with RSSI was conducted, which found RNSI to be significantly more accurate in a hospital environment with high signal interference. RNSI accuracy was 83.3%, whilst RSSI accuracy was 51.9%. Fingerprinting was excluded, and the RSSI method was proximity-based, with no mitigation for multipath effects.

**Figure 10:**
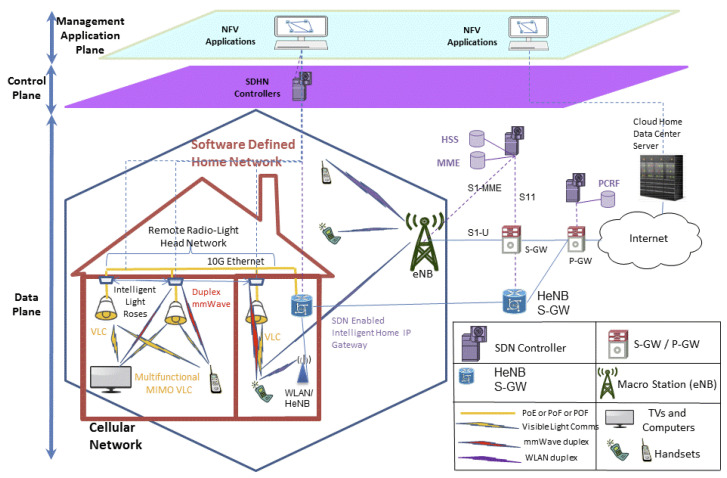
IoRL network architecture. Reprinted with permission from [96].

The Internet of Radio Light (IoRL) project [94] is being developed to integrate 5G-compatible mmWave remote radio heads (RRH) and VLC within the ceiling lights of rooms to provide an indoor broadband communication network with a location accuracy of less than 10 cm. The combination of VLC and mmWaves lowers the energy consumption at the Tx/Rx, reduces electromagnetic interference and mitigates the errors from NLoS signals [95]. Figure 10 shows the IoRL network architecture.

### 3.5. Examples of Indoor Localisation Systems Used within the Healthcare Domain

We found a lack of studies that aimed at monitoring patients in real-life scenarios or that related indoor localisation data with clinical outcomes. Most of the literature is focused on technological developments of the systems. Two studies were found that were deployed to monitor a group of patients, both using RSSI with BLE. The systems took different approaches, as one was deployed in a premapped rehabilitation centre and the other one was used in patients’ homes which are not mapped.

#### 3.5.1. Tracking Patients in a Post-Acute Rehabilitation Centre

**Figure 11:**
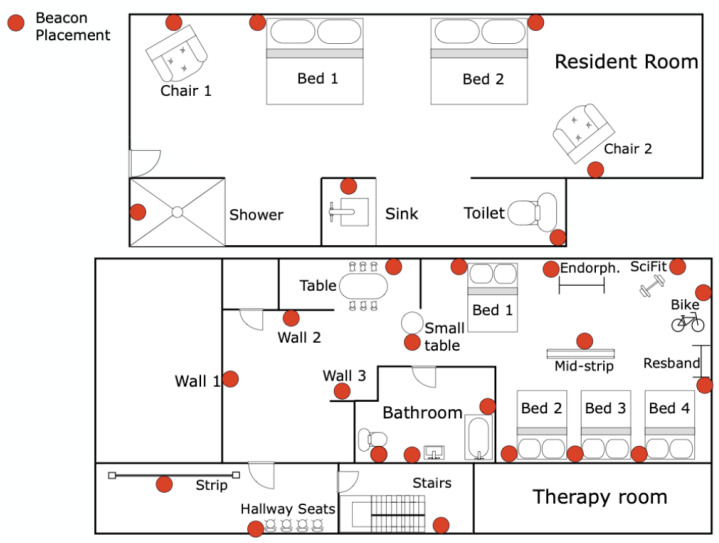
Map of the sub-acute rehabilitation facility: BLE beacon locations are represented by the red circles. Reprinted with permission from [26].

**Figure 12:**
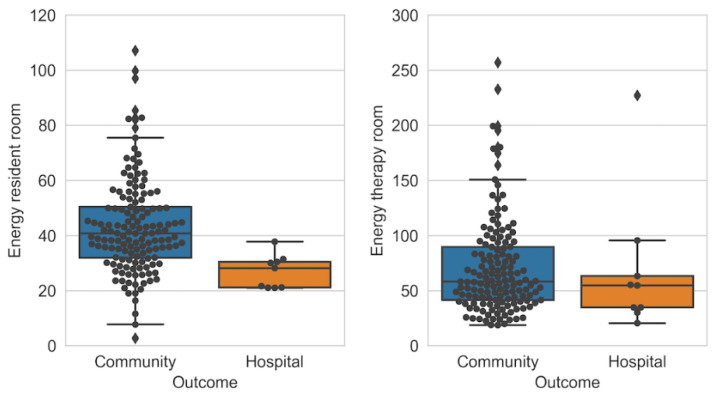
Energy intensity distribution amongst 2 outcome groups in the resident room and the therapy room. Reprinted with permission from [26].

Ramezani et al. [26] used BLE localisation along with accelerometer data to monitor 154 patients admitted into a post-acute rehabilitation facility for 21 days, after which their outcome was either being readmitted to the hospital or staying within community care. The study used proximity-based localisation through RSSI, with BLE beacons placed in locations of interest, as shown in Figure 11. A Sony Smartwatch 3 was used, which was worn by the patients from 9 am to 6 pm every day. Patients stayed in the resident room and spent 1 h in the therapy room for scheduled daily exercise. Features such as energy spent during walking or in bed and sitting time in bed were developed by combining accelerometer data for activity recognition and energy expenditure with indoor localisation to measure the time and energy spent in locations of interest. The features were compared with the outcomes using chi-square and Kruskal–Wallis tests, and the most significant features were used to develop a predictive model using random forests. The three most significant features were standing time, laying down time and resident room energy intensity. Therapy room energy intensity, in comparison to resident room (Figure 12), was not as significant, highlighting the importance of measuring energy intensity during free living (resident room). The ability to use the toilet was also a significant factor, and was measured manually through an activities of daily life (ADL) evaluation, as well as through BLE localisation and accelerometer data by measuring energy intensity in the resident bathroom. It is important to note that the hospital outcome group only consisted of 9 out of 154 patients, and a larger group would be required to produce meaningful conclusions.

#### 3.5.2. Vesta: Tracking Patients Undergoing Heart Valve Surgery

**Figure 13:**
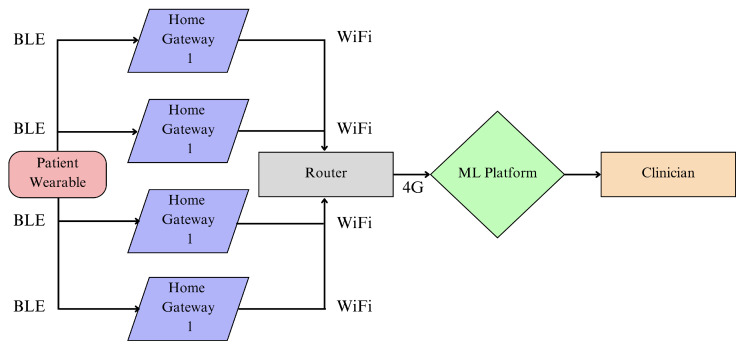
Vesta platform overview. Reprinted with permission from [72].

McConville et al. [72,97] developed a smart home system called Vesta, used to monitor the activity of 40 patients in their homes before and after heart valve surgery. Vesta uses a custom wrist-worn wearable containing an accelerometer and a BLE beacon, which sends data to four gateways installed around a home, each containing a Raspberry Pi. The gateways listen for BLE advertisement packets, which contain accelerometer data and have an RSSI that is unique to each gateway, used to locate the user. The four gateways connect to a 4G router through WiFi, which sends the data to be analysed. An illustration of the system is shown in Figure 13. The system is designed to be easily installable by a nontechnical user. It requires the user to plug in the four gateways around their home, in the living room, bedroom, kitchen and a custom room, followed by a short fingerprinting training phase.

**Figure 14:**
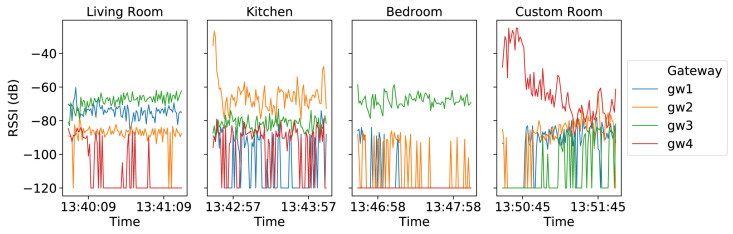
Fingerprinting training phase to achieve room-level localisation using RSSI at 4 gateways. Reprinted with permission from [72].

The study provides four health indicators that are selected after consultation with clinicians: time spent walking in different stages of care; time spent in rooms and number of transfers between rooms; time spent outside the home; quality and quantity of sleep. Activity recognition via the accelerometer is classified into walking, lying and sitting. For localisation, the user is instructed to sit in the living room, walk in the kitchen, lie down in the bedroom and conduct a typical activity in the custom room. The RSSI at each gateway is used to create fingerprints for room-level localisation; an example is shown in Figure 14. From Figure 14, it can be seen that the RSSI at gateway 3 is very similar inside the living room and the bedroom. The room can still be easily distinguished by leveraging the RSSI from all four gateways.

**Figure 15:**
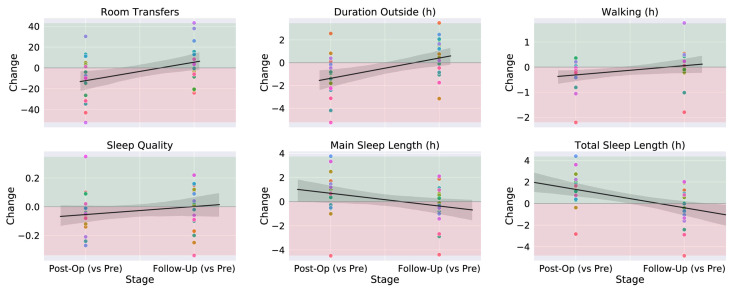
Graph comparing the measurements obtained from activity recognition and room localisation during the preoperation, postoperation and follow-up phases. Each patient is represented as a coloured point. Linear regression line demonstrates the patient recovery trajectory. Reprinted with permission from [72].

The localisation data assist in the validation of sleep detection, which is measured through the accelerometer. A room transition would negate an accelerometer outputting data that matches the pattern of sleep. In a similar way, the detection of room transitions requires the accelerometer data to detect walking in order to reduce false transitions. The health indicators measured for 20 patients can be seen in Figure 15. The Post-Op column compares the change from measurements during the preoperation phase to measurements in the first 2 weeks after the operation, with results being as expected due to the recommended rest immediately after the operation. Sleep length increases but the sleep quality is reduced, possibly indicating discomfort or pain. The Follow-Up column compares changes from measurements during the preoperation phase to measurements in weeks 12–16 after the operation, where the patient is expected to have recovered and benefitted from the surgery. These results are also expected; the activity levels and sleep quality rise compared to the preoperation phase and the postoperation phase.

**Figure 16:**
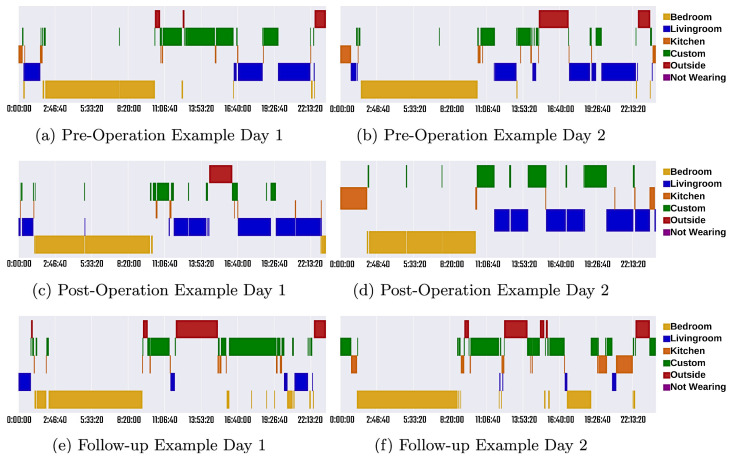
2-day room localisation data for Patient A in the different phases of their operation. Reprinted with permission from [72].

Detailed activity reports for two patients with different experiences are also provided in the study for a detailed analysis. Patient A underwent mitral valve repair, tricuspid valve repair and coronary artery bypass graft, and reported an improvement in wellbeing. Figure 16 gives an illustration of their room data for 2 days in the different stages of their operation. It demonstrates how the daily life of a patient can be recorded and how by collecting data over a period of time, any changes that occur can easily be detected.

**Figure 17:**
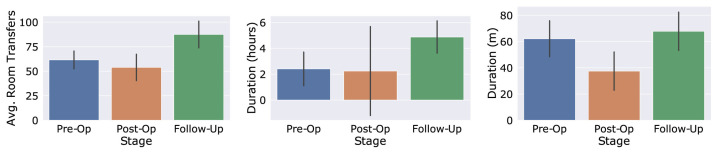
Detailed information on the health indicators for Patient A during the different phases of their operation. Reprinted with permission from [72].

Figure 17 shows the detailed health indicators of Patient A. Daily room transfers, time spent outside and walking duration all increased in the follow-up phase, indicating a successful operation, resulting in an increase in health and mobility. The information obtained from clinical notes and patient-reported outcomes for Patient B included that after the operation, they required a walking aid, and their restless leg syndrome worsened. Their sleep also became fragmented, and they still had symptoms that required rest during the day. This is supported by the in-depth data available for Patient B, which can be found in [72].

The data collected are validated through patient-reported outcome measures (PROMs), which is a questionnaire conducted once before and once after their operation. PROMs also rely on the patients’ memory, and are subjective. Vesta enables quantitative measures of health and mobility over a large period of time, and allows the data to be visualised in a useful way for clinicians. This provides a quantified measure of the impact that a medical intervention has on the daily life of a patient, and measures the effectiveness of the surgery.

## 4. Physiological Parameters

Physiological parameters such as heart rate, blood pressure and body temperature are commonly measured in clinical settings. These parameters vary greatly for each individual depending on internal and external factors and during daily activities. Infrequent measurements of these parameters, only when a patient is exposed to a clinical setting, means they can only be compared to the average measurements of a population and their own limited recordings. Continuous or frequent monitoring during free living allows one to establish a baseline for an individual, which can be used to measure changes over time and produce patterns [98]. Combining these parameters with external factors data obtained from other sensors in the IoT ecosystem can enable the development of complex systems.

There are two key sensors available on consumer wearables that can obtain information about an individual’s cardiac cycle and haemodynamics; the electrocardiogram (ECG) and photoplethysmography (PPG).

### 4.1. Electrocardiogram

An electrocardiogram (ECG) is a recording of the electrical activity of the heart. It is widely used in clinical settings to detect cardiovascular conditions such as heart attacks and rhythm disturbances that change the heart’s electrical activity. An ECG is measured by placing electrodes on the chest and limbs of a patient, which record different views of their heart’s electrical activity, with each view being labelled as a ‘lead’. A conventional ECG consists of 12 leads, which provide a full overview of the heart’s electrical activity. Six of the leads are obtained from placing four electrodes, one on each limb, providing leads I, II, III, VL, VF and VR. Six electrodes are placed on the chest, providing leads V1–V6 [99].

**Figure 18:**
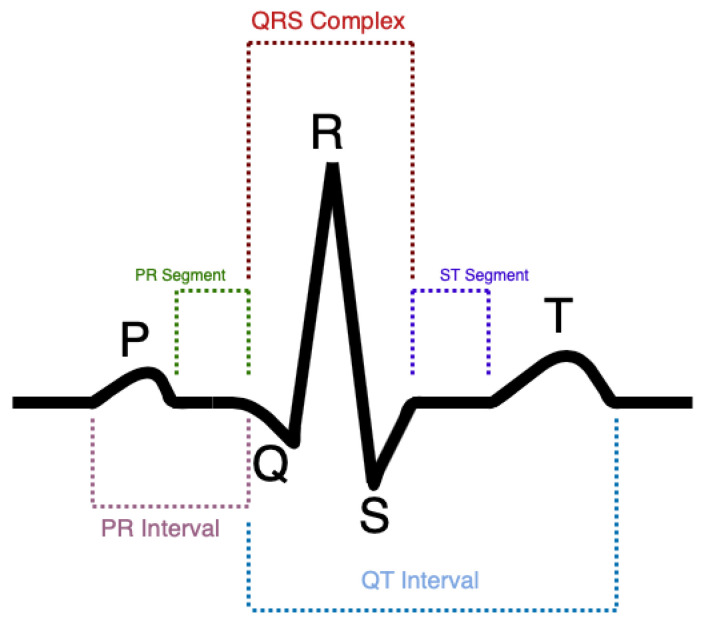
An ECG waveform, which is informative for the heart health conditions. Details about the PQRST can be found in [99].

An ECG waveform consists of five components and represents a single heartbeat, as illustrated in Figure 18. Various observations can be made from analysing this waveform. For example, an elevated ST segment can be caused by a patient with acute myocardial infarction (heart attack). A depressed ST segment can be caused by ischaemia (reduced blood flow). An older myocardial infarction can cause the QRS to start with a deep downward deflection [99].

**Figure 19:**
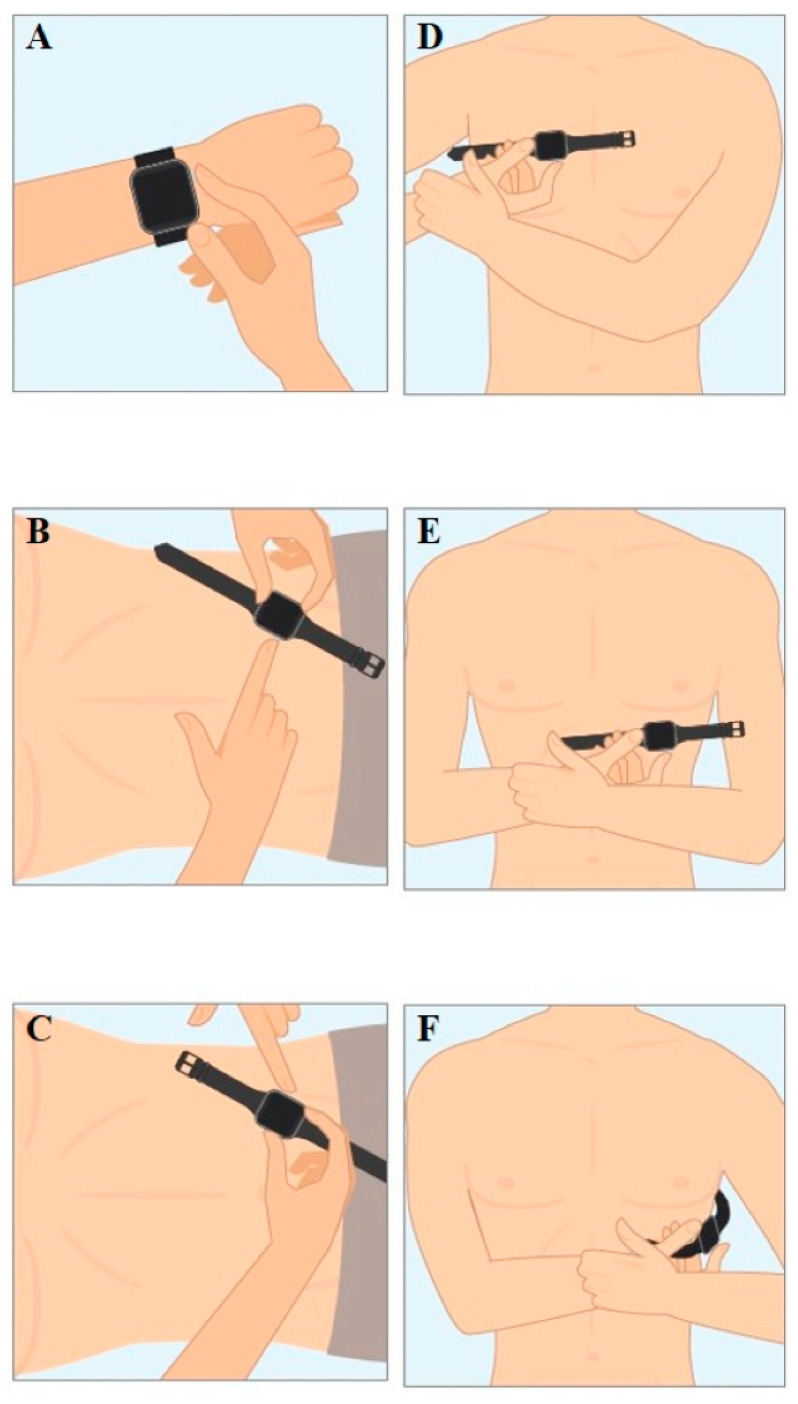
Illustration of a participant taking ECGs using an Apple Watch to obtain different leads. (**A**) I, (**B**) II, (**C**) III, (**D**) V1, (**E**) V4, (**F**) V6. Reprinted with permission from [101].

**Figure 20:**
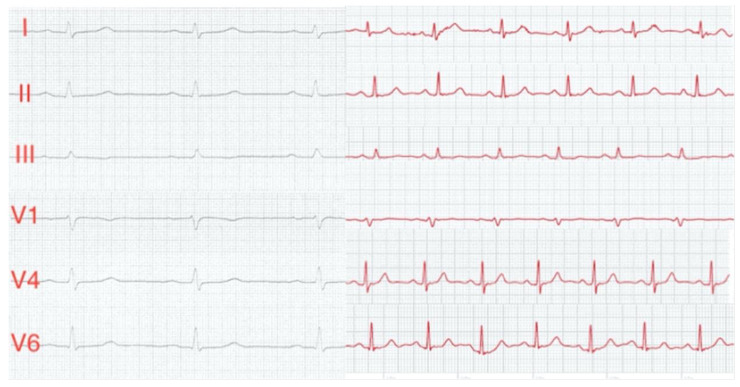
Comparison of 6 leads between the Apple watch (red) and the standard 12-lead ECG (black). Reprinted with permission from [101].

Clinicians often use ambulatory ECG monitoring if symptoms occur randomly throughout the day. They apply three electrodes to a patient’s chest, connected via wires to a recorder on the waist for 24–48 h [100]. It is not suitable for long-term monitoring due to its obtrusive nature. However, implantable recorders that are injected under the skin can provide long-term ECG recordings for years. Recent advances in wearable devices have seen ECG electrodes incorporated within some consumer smartwatches. Electrodes are usually placed in the back of the smartwatch in contact with the wrist and the crown, which a user is required to touch with their finger. This orientation provides lead I if the watch is worn on the left wrist. A study by Samol et al. [101] used the Apple Watch Series 4 for 50 healthy participants to measure their ECG readings. The study asked users to place the back contact of the watch at various positions on their bodies corresponding to the leads whilst placing the left or right index finger on the crown electrode (Figure 19). Users were able to obtain six leads: I, II, III, V1, V4 and V6. These recordings were compared against the 12-lead ECGs to determine the feasibility of using a smartwatch for easily accessible, patient-directed ECGs. The ECGs obtained from the smartwatch had a good signal quality, usable for diagnostics, and were identical to those obtained from the standard 12-lead ECGs (Figure 20). Three cardiologists were asked to match the smartwatch leads to the standard 12-lead; leads I, II and III were matched with 100% accuracy, whilst V1, V4 and V6 had 92% accuracy. Two patients with acute anterior myocardial infarction were also included in the study. The cardiologists were able to correctly diagnose from the elevated ST segment in the smartwatch ECGs.

The KardiaMobile device by AliveCor is an FDA-approved handheld device that can provide a six-lead ECG and can detect atrial fibrillation, bradycardia, tachycardia or normal heart rhythm. It has an accompanying smartphone application that stores and displays the results of the ECG transmitted via Bluetooth. Users can also see a visual summary of their recordings and can easily share their ECG with a physician [102]. KardiaMobile has been used in multiple studies to remotely monitor patients [103,104,105].

### 4.2. Photoplethysmography (PPG)

**Figure 21:**
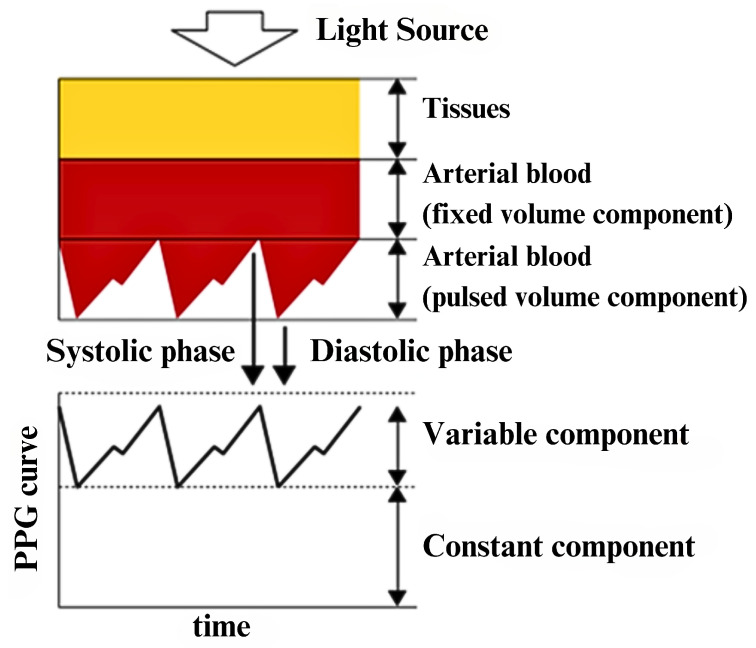
PPG mechanism. The graph at the bottom gives an example of the raw signal obtained from PPG and how it corresponds to the flow of blood in the artery. In the systolic phase, there is less blood volume, so less of the light is absorbed, and hence, it gives a larger signal. Reprinted with permission from [107].

Photoplethysmography (PPG) is a low-cost optical technique that uses an LED to emit light onto the skin that passes through tissues and is significantly absorbed by blood. The reduced-intensity light is measured by a photodetector to determine the blood volume variations during circulation [106,107]. An illustration is shown in Figure 21.

**Figure 22:**
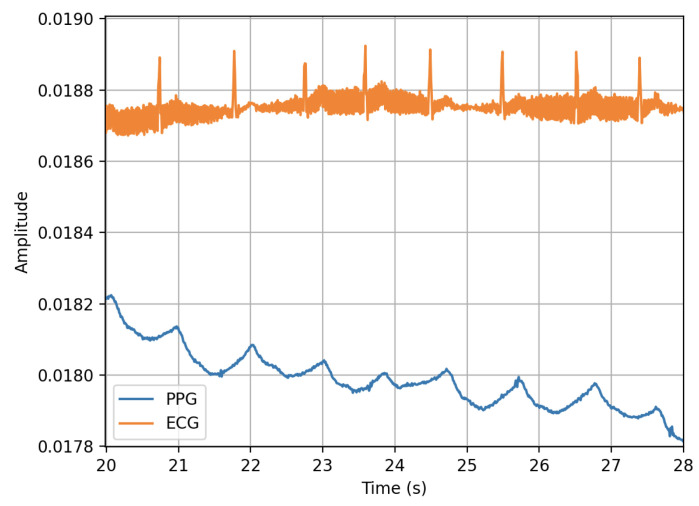
A single-lead ECG and PPG collected at the same time by a smartwatch on the wrist. The PPG waveform peak comes after the ECG waveform peak.

PPG measures the pulse rate, which is the rate of change in blood pressure due to the ventricular ejection of blood [108]. The pulse rate corresponds to the heart rate, which is the rate of heart contraction. Figure 22 shows a single-lead ECG and PPG waveform taken by a smartwatch at the same time. The peak of the PPG waveform slightly lags behind the R complex of the ECG waveform due to the time taken for the blood flow to the tissue after the electrical impulse. This lag is called the pulse transit time (PTT). The peaks from the two waveforms have a high correlation coefficient, allowing PPG to be used to analyse the R-R interval [109].

PPG can be used in two orientations: transmission and reflective. In the transmission orientation, the photodetector is placed opposite the LED, measuring the light that passes through the tissue. In reflective orientation, the photodetector is placed next to the LED, measuring the reflected light. The transmission signal provides a better signal quality; however, the placement is restricted, and it becomes obtrusive [107]. Reflective orientation is commonly found in the back of smartwatches that measure from the top of the wrist. This enables passive recordings to be collected from the PPG sensor without any user intervention. Additionally, PPG can be used in a contactless format by using a camera to measure the colour changes on the skin caused by blood flow [110]. Hu et al. [111] used a laptop camera to measure the heart rate of multiple people simultaneously using variations in facial skin colour. PPG sensors in smartwatches have been validated in several studies for their accuracy in providing heart rate measurements to an acceptable level against the gold-standard 12-lead ECG method [108,112,113]. Inaccuracies are generally higher during motion, and vary amongst devices due to different sensor hardware and algorithms used to compute HR values from the raw PPG signals [114].

### 4.3. Heart Rate

**Figure 23:**
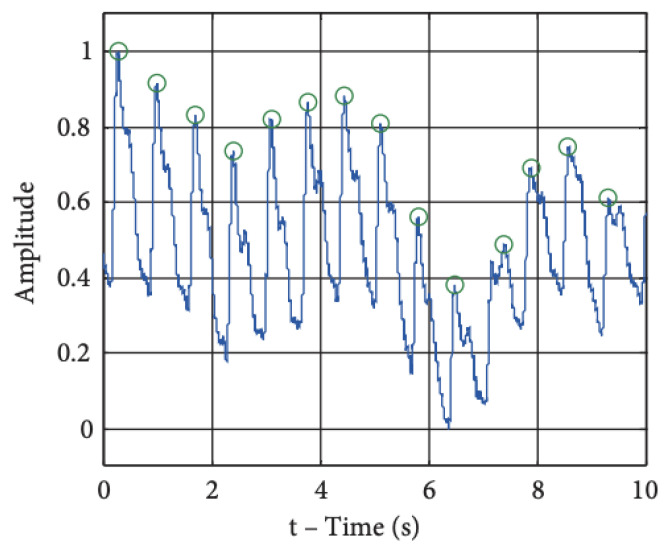
Peak detection algorithm used on a raw PPG signal to calculate HR. Reprinted with permission from [119].

Heart rate (HR) is a vital sign of health, and is measured in beats per minute (BPM) [115]. HR is influenced by a variety of factors, such as age, activity, disease, emotions and medications [116]. Continuous heart rate monitoring can provide a plethora of valuable information about an individual’s body in different contexts, and can be combined with data obtained from other sensors to observe more complex phenomena [117]. HR on wearable devices is usually measured using a photoplethysmography (PPG) sensor, which is the most common sensor after the accelerometer in consumer wrist-worn wearables [118]. A simple peak detection algorithm can be used to extract the heart rate from a raw PPG signal; the peak-to-peak waveform represents 1 cardiac cycle (Figure 23) [119]. A recent relevant example of wearable-device HR monitoring includes the detection of COVID-19 and other respiratory viruses before the onset of typical symptoms or in asymptomatic cases, as these infections have been associated with an elevated HR [120,121].

### 4.4. Heart Rate Recovery (HRR)

**Figure 24:**
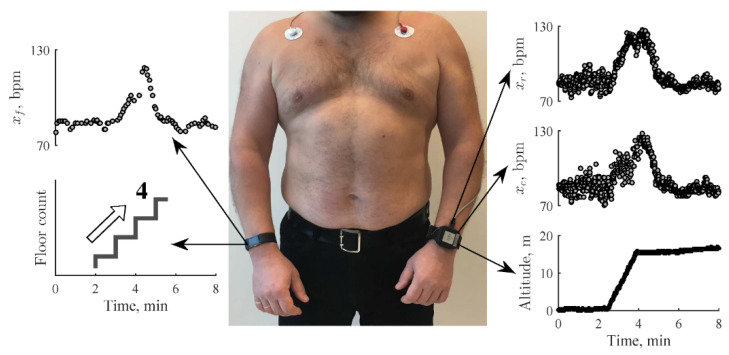
Custom wearable with PPG and barometric pressure sensors providing values for heart rate and altitude climbed over time. An ECG is also acquired (left arm). The right arm uses a consumer device, Fitbit Charge 2, which provides the HR and floor count. Reprinted with permission from [123].

A cardiac-related example includes the monitoring of heart rate recovery (HRR), which is the rate at which the heart rate decreases immediately after stopping physical exercise. A meta-analysis by Qiu et al. [122] concludes that a slow HRR is associated with an increased risk of cardiovascular events, and recommends the recording of HRR for risk assessment as a routine clinical practice. Sokas et al. [123] developed a custom wrist-worn device that includes a PPG sensor to measure heart rate and barometric pressure sensor to measure altitude. The wearable is designed to measure the decay in heart rate after a user climbs a set of stairs to obtain the HRR, in a study of 54 healthy participants at different climbing rates. The rationale to measure HRR through stair climbing is that it is a common daily activity usually followed by rest or reduced activity, and can be performed easily in free-living conditions. Participants climbed 4 floors of 96 stairs in total, at a climbing rate of 48, 72 and 96 steps per minute. An example dataset can be seen in Figure 24.

**Figure 25:**
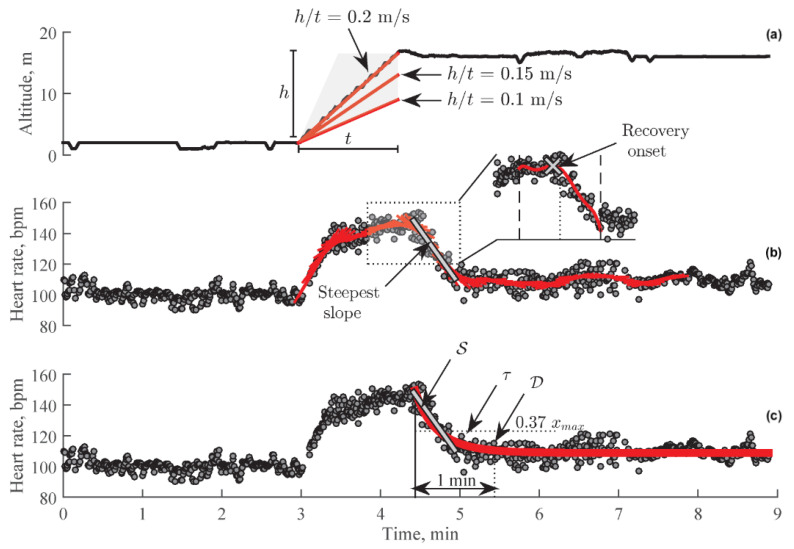
Detection of HR recovery onset and parameter extraction. (**a**) Altitude as measured by the barometric sensor, (**b**) detection of the steepest falling slope corresponding to the recovery onset, (**c**) estimation of HRR parameters. Reprinted with permission from [123].

After stair climbing is detected via the barometric sensor, the recovery period search is performed by fitting a linear polynomial to the heart rate data in a sliding window of 1 min. The time interval with the steepest downward slope is determined to be the recovery period. Heart rate data 25 s before and after the onset point is used to obtain the maximum HR value through fitting a sixth-order polynomial. An illustration can be seen in Figure 25.

**Figure 26:**
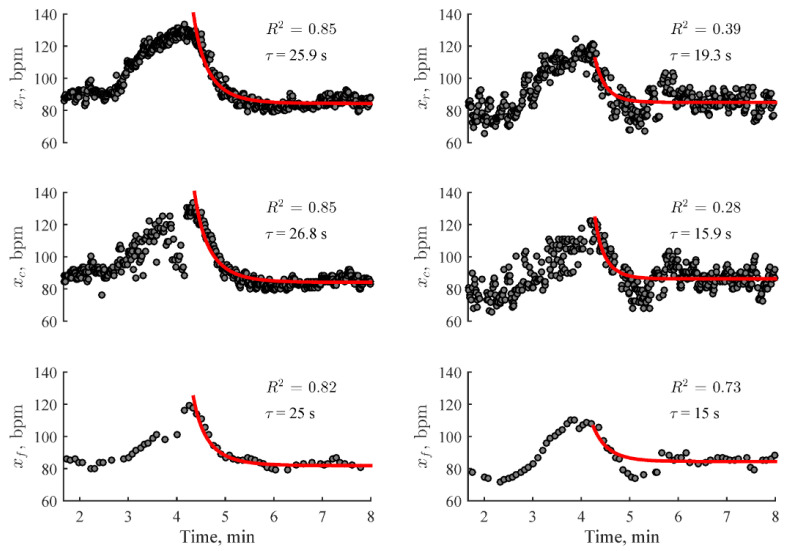
Examples of exponential fittings. Slower HRR recoveries are displayed on the left column, which yield a higher coefficient of determinant. Faster HRR recoveries are displayed on the right column; there is a higher heart rate variability in the slower recovery phase, which results in a lower coefficient of determinant. Reprinted with permission from [123].

The fastest recovery period occurs immediately after the end of the physical activity and then slows down. It can be approximated using the following monoexponential model in (4) [124]:

(4) $$x_{m}\left(t\right)=x_{0}+\Delta xe^{-1/\tau },$$

where $x_{0}$ is the HR at the end of the recovery phase; Δx is the difference between HR at the start and end of the recovery phase; and τ is the time constant of the exponential decay calculated from Figure 25. Examples of exponential fittings from the study are illustrated in Figure 26.

Stair climbing is unlikely to cause HR to reach the intense zone; hence, HR recovers faster when compared to intense activities, which increases the heart rate variability in the slower part of the recovery period. This leads to a reduced coefficient of determinant (R2), as displayed in the right column of Figure 26. Bartels-Ferreira et al. [125] also found significantly lower R2 values when measuring HRR after low-intensity activities compared to when measuring after high-intensity activities. The study described in this section used only a barometer sensor to detect a period of moderate-intensity physical activity to measure HRR. Wearable devices can make use of other sensors such as accelerometers to automatically measure HRR after a period of intense activity using the same modelling techniques used, which would yield a higher coefficient of determinant.

### 4.5. Resting Heart Rate (RHR)

**Figure 27:**
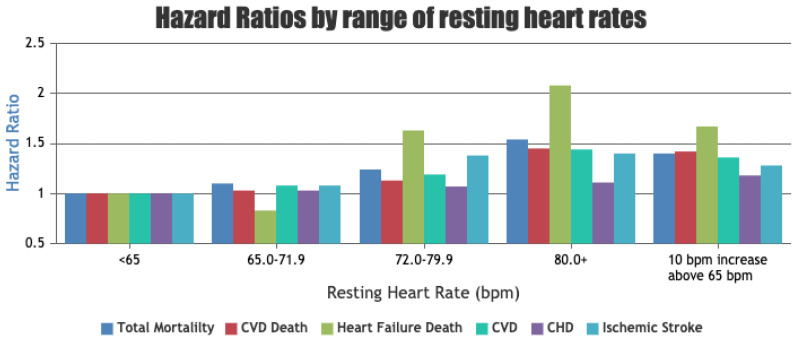
Hazard ratios for CVD-related outcomes based on RHR ranges compared to a reference group of individuals with RHR <65 bpm. Graph produced with data from a study by Woodward et al. [126].

Resting Heart Rate (RHR) is the HR of an individual in a state of complete rest whilst awake, ranging from 60 to 80 bpm for an average human [112]. A low RHR generally indicates better cardiovascular fitness and reduced risk of various CVDs. Figure 27 shows the relative risk of death from different causes with relation to the RHR.

A state of the art by Fox et al. [127] goes into extensive detail on the relation between RHR and various CVDs, with most findings indicating a high RHR to have an increased general risk. We will not go into further detail, but it can be concluded that RHR is an important health indicator that is easily measurable by consumer smartwatches using PPG. A study by Dunn et al. [128] demonstrated that RHR collected by a smartwatch provides more consistent measurements compared to collections in the clinic, due to being able to collect RHR after a longer period of inactivity. Fitbit devices with PPG sensors display a visual graph, plotting the trends in RHR over time in the accompanying mobile app. RHR is usually determined by measuring the HR in the morning when the user wakes up [129]. A decreasing RHR without an obvious cause such as exercise can have negative implications. A patient who noticed a gradual decline in her RHR, measured via Fitbit, over a 2-month period was diagnosed with a 2:1 atrioventricular block after investigation [130]. This further highlights the need to monitor physiological parameters over a long period of time, as unusual changes can be associated with disease.

### 4.6. Energy Expenditure Using HR

**Figure 28:**
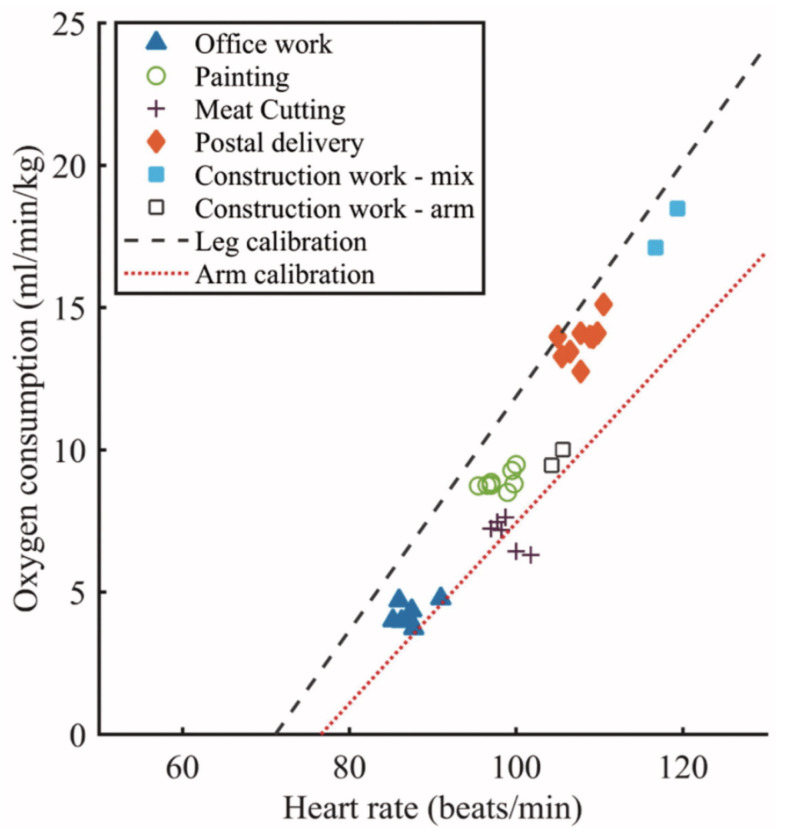
Relationship between HR and VO2 during different daily work tasks. Reprinted with permission from [135].

Total energy expenditure (TEE/EE) consists of EE during physical activity (PAEE), EE during rest (REE) and the thermic effect of food (TEF), and can be measured in either kilocalories (kcal) or kilojoules (kJ) [131]. Oxygen consumption (VO2) can be directly used to measure EE, with approximately 5kcal of energy expended for every litre of oxygen consumed [132]. Physical activity, which can be quantified in terms of intensity [133], increases VO2, and results in the expenditure of energy; hence, measuring EE allows for the monitoring of physical activity. During exercise, HR and VO2 are linearly related; therefore, measuring HR can be used to estimate EE, and in turn, the pattern of physical activity, including intensity, frequency and duration [134]. A study by Yang et al. [135] measured HR and VO2 during tasks for different professions with varying physical activity levels. The correlation between them is displayed in Figure 28.

This allows for an in-depth analysis of an individual’s physical activity, including free-living activities and activities that are nonrhythmic and may not be accurately measured on a step counter. However, using HR on its own to infer physical activity can lead to many errors, due to HR and VO2 not being linear during rest and low intensity activities, and variations in HR due to internal and external factors such as illness, stress and caffeine [136]. Combining HR measurements with movement registration using accelerometry significantly improves the estimate of PAEE. The combination of sensors to measure EE also factors in any additional load on the user, which would be completely disregarded using an accelerometer alone [137]. In a study by Kuo et al. [138] accelerometer values had very few differences when participants walked at the same speeds but at different gradients. Incorporating Δ HR (exercise HR – RHR) within their linear/multiple regression equations to estimate EE yielded a higher coefficient of determination. A similar study was performed by He et al. [139], which compared the difference between HR and accelerometer EE measurements for increasing levels of incline on a treadmill at the same speed. Measured EE increased with increasing incline using HR measurements, but did not increase using accelerometer measurements.

#### 4.6.1. PAI—Personalised Activity Intelligence

Nes et al. [140] derived a metric of physical activity measurement that is assessed on a weekly score system (PAI) and obtained through continuous HR monitoring. Although PAI does not explicitly use EE, it builds on the same concept of the relationship between HR and VO2. A PAI score of 0 refers to an inactive group, and achieving a PAI level of 100 or more has a reduced risk of cardiovascular disease mortality by 17% and 23% in men and women, respectively, compared to the inactive group. This metric characterises the HR that measures the body’s response to activity instead of just measuring physical activity, validated by participants who reached the physical activity recommendations but still had a higher risk of CVD mortality (compared to the <100 PAI level group) as they did not reach a PAI level of 100. This provides a more personalised activity target when compared to a general steps target, for example. A PAI level of 100 or more corresponded to 40 min of high-intensity or 60 min of moderate-intensity activity every week. The intensities (K) were 85% and 75% for high and moderate intensities, respectively, and were worked out using the heart rate reserve method, (HRmax − RHR)K + RHR [141]. The derivation cohort consisted of 4631 healthy participants from the HUNT Fitness Study [142,143], whose VO2 peak was measured on a treadmill running test. The validation cohort for the analyses had 70,535 participants. PAI can be used on smartwatches that provide continuous HR monitoring, including Apple Watch, Fitbit, Zepp, Polar and Amazfit devices [144].

#### 4.6.2. Beta-Blocker Patient Model

Cardiac patients are often prescribed with beta-blocker medications, which aim to treat CVDs and prevent cardiac events by reducing HR and blood pressure during exercise [145]. An EE estimation model using HR that was developed for the general population is ineffective for use with patients who are regularly using beta-blockers. as their lowered HR during exercise will result in the model not registering the activity or underestimating it. Kraal et al. [136] conducted a study with 16 cardiac rehabilitation participants on beta-blockers, which measured their EE during various activities (walking, vacuuming, cycling, etc.) in order to derive an EE prediction model that factors in the effect of beta-blockers using a combination of HR and accelerometer data. The study used multivariate linear regression analyses to develop the EE models which allowed for the iterative selection of independent variables to include in the models. The three independent variable groups used were (i) patient characteristics: age, weight, height, BMI, resting metabolic rate (RMR), beta-blocker dose and peak VO2; (ii) body movement, obtained from accelerometer data; (iii) HR and HRnet (HR-RHR)—five models were derived using a combination of the variables above, using ii (model a), i + ii (model b), iii (model c), i + iii (model d) and i + ii + iii (model e). Model e, as expected, had the highest coefficient of determination, at 0.76. It is important to note in this study that comparison models separating out the beta-blocker dosage from the patient characteristics were not developed; hence, the effect of the medication on the EE estimation cannot be determined.

Constant measurement of EE enables patterns and trends to be developed for a user, which can potentially provide predictions for patient outcomes, especially when combined with other data such as indoor location [26], as previously described in the indoor localisation section. Measuring EE through HR can also provide more accurate monitoring of exercise- based cardiac rehabilitation programmes such as circuit weight training (CWT), where using higher weights results in increased EE [146,147].

### 4.7. Heart Rate Variability (HRV)

**Figure 29:**
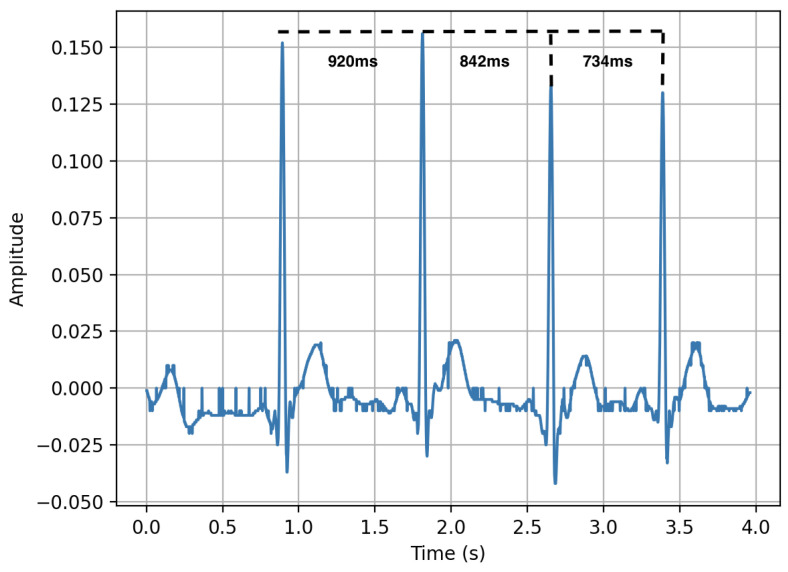
Snapshot of an ECG recording with the 3 R-R intervals measured and labelled, displaying the variability between each heartbeat.

Heart rate variability (HRV) measures the time intervals between each heartbeat. It can be extracted from the R-R interval from an ECG waveform, as illustrated in Figure 29, and from the peak-to-peak interval from a PPG signal. A high HRV is generally considered healthy [148].

There have been many studies that highlight the importance of HRV as a health indicator. A study by Kleiger et al. [149] on 808 patients found a strong correlation between low HRV and mortality after acute myocardial infarction, with the relative risk of mortality being 5.3 times higher in the group of patients with HRV of less than 50 ms, compared to the group of patients with HRV of more than 100 ms. A study by Kotecha et al. [150] measured the HRV over 5 min via a 12-lead ECG for 470 patients undergoing diagnostic angiography for obstructive coronary artery disease (CAD). Power spectral density analysis was used to deconstruct HRV into its component frequencies, with the low-frequency (LF) power found to be inversely related to the extent of CAD. A cut-off value of 250 ms^2^ was identified as a significant independent predictor of CAD within the cohort.

### 4.8. Blood Pressure—Ambulatory BP

**Figure 30:**
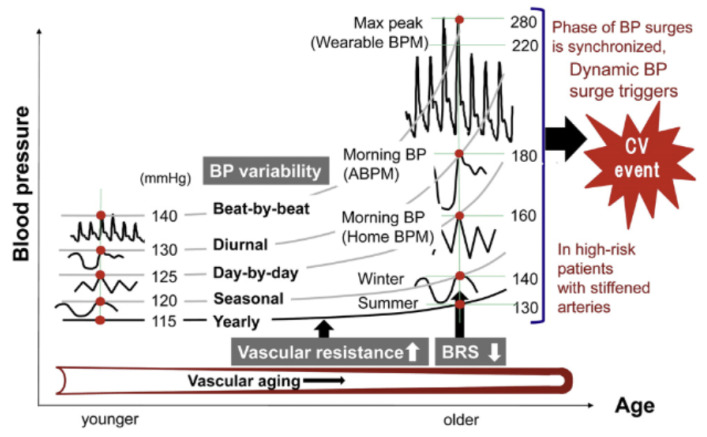
The variability of blood pressure caused by different factors. Reprinted with permission from [152].

Blood pressure (BP) is an important indicator for cardiovascular health, with high BP being a major risk factor for CVD, and the commonest preventable cause of death worldwide [151]. BP varies throughout the day, and is affected by external factors such as stress, weather and sleep. Figure 30 provides some detail on the factors that affect BP.

BP measures two values: systolic pressure, the peak pressure during cardiac contraction; and diastolic pressure, the pressure when the heart relaxes and refills between beats [153]. BP is typically measured using an inflatable cuff that wraps around the arm. The cuff inflates until it is tight and cuts the blood flow, after which it deflates. As the cuff reaches systolic pressure, blood begins to flow, causing vibrations that are recorded by the monitor. The cuff continues to deflate until the vibrations stop, reaching the systolic pressure [154]. Traditional cuff-based systems are bulky and are unsuitable to be carried by a user. Various devices have been developed that have condensed the BP measurement into a portable system with Bluetooth capabilities to record data on a smartphone. The HeartGuide smartwatch by OMRON [155] integrates the inflatable cuff under the straps of the smartwatch, and a BP measurement can be initiated by the user.

#### PPG for BP Monitoring

The use of a PPG sensor to measure BP is an emerging technology [11]. PPG is a cuffless system that is less invasive and can continuously record signals. The pulse transit time (PTT) has shown to be inversely proportional to BP [156]. PTT was previously described in the PPG section; it is measured by measuring the time difference between an ECG and PPG peak. Simultaneous PPG and ECG monitoring has shown potential to monitor BP in a cuffless manner [157]. There is a lack of thorough validation studies, and any readings from a PPG system should be interpreted cautiously.

### 4.9. Sleep

Sleep health can be characterised in terms of measures such as quantity, continuity and timing. Sleep health is associated with various health outcomes and is a key indicator of health and wellbeing [158]. An example of this is described in Section 3.5.2, where a patient had fragmented sleep due to a worsened condition postsurgery. Buysse [158] provides a summary of key studies that have been published that associate health outcomes with measures of sleep. Notable examples include the association of sleep quality, timing and duration with coronary heart disease [159,160,161].

**Figure 31:**
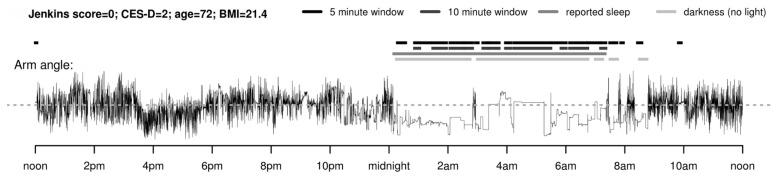
Changes in arm angle over time, measured by an accelerometer. The significantly thinner line after midnight indicates sleep. Reprinted with permission from [162].

Van Hees et al. [162] developed an algorithm to detect sleep from a wrist-worn accelerometer by estimating the arm angle relative to the horizontal place. The sleep period is characterised by low frequency of changes in the arm angle, illustrated in Figure 31.

A study by Renevey et al. [163] measures sleep in more depth, estimating the different phases of sleep, rapid eye movements (REM), non-REM (NREM) and awake time. In addition to using the accelerometer to measure motion, the study uses a PPG sensor to analyse HR and HRV to assist in determining sleep phases. The use of a PPG sensor provides further insight into sleep health, such as detecting sleep apnoea through oxygen saturation data [164,165].

### 4.10. Patient-Reported Outcome Measures (PROMs) and Ecological Momentary Assessment (EMA)

Patient-reported outcome measures (PROMs) are widely used in clinical settings to obtain subjective information about a patient’s health and wellbeing. Questionnaires vary significantly based on the indication for use. A basic example is a question on the presence of symptoms, with a follow-up enquiring about the severity of the symptom, which could be based on a scale of 1–10 [166,167]. PROMs are usually only recorded when a patient comes into a clinical setting, which may be very infrequent. Describing a symptom from the past also relies on the memory of the patient. Kheirkhahan et al. [27] developed a smartwatch system that prompts a user every day to complete a questionnaire that measures the wellbeing of the patient, such as pain on a numerical scale, mood and fatigue. This essentially constructs a symptom diary, which can be presented to a physician instead of recalling symptoms from the last clinical visit. Collecting PROMs on a wearable device with other sensors also provides the opportunity to extract sensor data when the patient reports a decrease in wellbeing. For example, a patient can be prompted to immediately record an ECG on their smartwatch if they enter chest pain as a symptom. Additionally, PROMs can be used to validate the data that is collected by sensors, demonstrated by the Vesta system described previously [72].

## 5. Where Might Such Technologies Fit into Cardiovascular Healthcare?

The ability to intermittently or constantly monitor patients in their daily lives and homes is relatively recent, and is driven more by societal technological trends (smartphones, WiFi, etc.) than by healthcare need. However, given their potential, it is essential for healthcare to adapt to these new technologies. We describe here how such technologies may be applied to several common cardiovascular diseases, as well as the implications this raises.

### 5.1. Arrhythmia

Many people experience intermittent (sometimes called “paroxysmal”) abnormal heart rhythms. The detection and monitoring of such rhythms is the area of cardiovascular medicine that has benefitted the most from the technologies we describe, in part because an ECG during an abnormal rhythm is usually the only type of data required to diagnose and monitor the underlying problem. The implications and risks of abnormal heart rhythms vary widely. Almost every adult will experience occasional “ectopic” beats, where a single heart beat is initiated in a different part of the heart, but these are rarely of any consequence. Around 1% of adults and 25% of the elderly develop atrial fibrillation (AF), which causes a permanent or intermittent (paroxysmal) irregular heart rate that is usually fast. This often does not cause any symptoms, but may cause breathlessness, dizziness or an awareness of an abnormal heart rate (sometimes referred to as “palpitations”). The main risk of atrial fibrillation is that it increases the risk of stroke due to embolisation of blood clots that form in the heart. Studies have already shown that devices such as the Apple Watch and Fitbit are able to detect AF, leading to FDA approval as medical devices.

Abnormal sustained heart rhythms that are initiated in the muscle of the ventricle are more dangerous, with ventricular tachycardia (VT) and ventricular fibrillation (VF) being common causes of sudden loss of consciousness and death without immediate defibrillation. Abnormally slow heart rhythms, usually caused by age-related degeneration in the heart’s conduction system, can cause dizziness, blackouts or death, and if detected, usually require a permanent pacemaker to be implanted. The detection of dangerous rhythms such as ventricular tachycardia (which may only last a few seconds before spontaneously reverting to a normal rhythm, such that the patient is unaware it has happened) requires drug treatment, and often the implantation of a defibrillator, which is life-saving if the patient later develops sustained VT.

Monitoring of the heart rate and rhythm in patients known to suffer or be at risk of intermittent or continuous arrhythmias [168,169] may serve several purposes. For example, in patients permanently in atrial fibrillation, monitoring the heart rate (either occasionally or continuously) at both rest and on exertion allows for the initiation or dose alteration of medications that reduce abnormally high heart rates. This does not reduce the risk of stroke (which is achieved by anticoagulant medication), but improves breathlessness and palpitations. This, in turn, may reduce the need for face-to-face consultations and more rapid identification of the correct dose and medication regimen for the individual patient.

Most paroxysmal arrhythmias occur relatively rarely in any patient; some only every few months or years. This means continuous monitoring may be required for years to be useful. Currently, the only realistic way to achieve this is by implanting a continuous ECG monitor, but this is invasive, and has a finite battery life. Wearable technologies (with appropriate regulatory approval as a medical device) that could more conveniently achieve the same level of ECG accuracy would be likely to replace many such implanted systems, although issues with interrupting recording to charge the device may limit use in potentially life-threatening rhythms such as VT.

### 5.2. Heart Failure

The efficiency of the heart’s pumping function can be affected by many factors, such as previous heart attack, genetic conditions, drugs such as alcohol or chemotherapy, high blood pressure, diabetes and obesity. Because many such factors are more common in older people, and because people with these conditions are living longer, heart failure has reached epidemic proportions, with over a million sufferers in the UK alone.

Heart failure causes breathlessness, limitation in physical activity, and swelling of the ankles and abdomen due to fluid retention (leading to increased body weight). Although some patients live with heart failure for many years, the outlook is poor, with half of patients dying within five years of diagnosis, often after multiple admissions, with worsening breathlessness and fluid retention, requiring lengthy stays in hospital for intravenous drug treatment, leading to very high healthcare costs. Early recognition of heart failure deterioration may reduce hospital admissions and improve outcomes, but unlike the detection of arrhythmia, there is no single form of data that accurately detects deterioration, requiring assessment of how the patient feels, body weight, oxygen saturation and other physiological parameters.

Because of its long-term nature, high risk of death and hospitalisation, and economic burden, heart failure is one of the most promising clinical applications for monitoring technologies. Multimodal data capture, including requiring patient input (answering questions about how they feel and their symptoms), measurement of physical activity, blood pressure, heart rate (which often increases as the condition deteriorates), body weight and novel factors such as speech quality may allow patients, families and clinicians to monitor the condition and initiate early treatment in the case of deterioration.

### 5.3. Valvular Heart Disease

A total of 2–3% of the adult population have valvular heart disease (VHD) caused by at least one leaking or narrowed heart valve [170]. Although it does not cause symptoms if mild, VHD usually deteriorates over years, eventually leading to breathlessness, which can be severe enough to cause hospital admission or death. The only treatment is surgical intervention to replace the valve, although newer procedures do not require open-heart surgery or cardiopulmonary bypass. VHD is diagnosed by ultrasound, and is often detected coincidentally in people at early stages of the disease when they have few or no symptoms. Such people are monitored for signs of symptoms and by intermittent ultrasound scans over years, but deterioration to the point of requiring surgery is still often missed.

VHD represents an currently unexploited opportunity for low-cost, unobtrusive monitoring that can be used over many years to detect eventual deterioration. This would reduce the chance of missing such deterioration, and may reduce the healthcare costs of periodic face-to-face clinical consultations. The most likely modality would be in measurement of physical activity, although multimodal monitoring that incorporates heart rate at rest and on exertion may provide more information on physiological changes associated with deterioration in valve function.

### 5.4. Practical Implications

Data are not clinically useful unless they change the decisions made by the patient or clinician in a way that improves outcomes for the patient. For any monitoring system to justify the investment required, there needs to be a clinical rationale for collecting such data, and any system then requires robust evaluation in properly designed clinical studies that quantify clinical and cost effectiveness. The 1:1 randomisation to placebo or active treatment used in drug trials is challenging to apply in technology studies, as neither patients nor clinicians are blinded to the intervention. However, other study designs, such as the stepped wedge cluster randomised trial, can be applied, in which hospitals are randomised to use the new technology in waves, and the effect of the implementation is measured.

## 6. Conclusions and Future Direction

This paper reviews literature from three different monitoring domains that can be implemented and enabled by wearables: physical activity monitoring, indoor localisation and physiological parameters. Existing technologies are described, and an overview is provided on how to monitor within these domains. Physical activity monitoring is widely utilised in existing consumer devices. Further analysis requires the use of raw data from accelerometers, gyroscopes and magnetometers, especially for the more frail population, where monitoring is more error-prone in consumer-level data that wearables provide, such as step count. Indoor localisation is complex, and there are multiple methods available to implement it. The selection of technologies and techniques is dependant on the requirements of the application, the main requirement being the level of accuracy. Methods combining technologies/techniques usually provide better performance. PPG and ECG sensors enable the monitoring of several physiological parameters, which are relevant to monitor for cardiac health based on the literature reviewed. Many of these parameters are monitored in consumer devices such as resting heart rate, which has proven to be useful in diagnoses. All three of these domains complement one another, providing enriched data when combined, and can be used to gain deep insight into an individual’s experience in relation to CVDs, ageing, physical activity and mobility.

During the literature search, many papers found either focused solely on the technology standpoint or on the clinical standpoint of CVDs. The significance of monitoring these domains from the perspectives of cardiac health is reviewed and highlighted throughout this paper to establish a clear link between the technological standpoint and the clinical standpoint, aiming to bridge the gap between the two.

### Future Direction

Monitoring technologies raise the question of who is monitoring the incoming data and what actions are taken in response. Most healthcare systems are ill-equipped to provide continuous clinical oversight of complex data streams; even in patients admitted to hospital, this is only achieved in the highest-intensity locations, such as coronary care and intensive care units. Implementation of more widespread monitoring of patients in the community is therefore only likely to be possible if parallel analytic systems are developed that interpret the data and automate the actions required in the case of alerts or changes that require medical interventions.

The integration of wearables and other IoT devices in monitoring cardiovascular diseases holds significant promise. However, future research will focus on long-term implementation, especially in diverse real-world scenarios to obtain longitudinal data. Furthermore, the development of sophisticated machine learning algorithms could enable more precise predictions of cardiovascular events, paving the way for timely and more specific interventions. Lastly, the effectiveness of these technologies should be tested in large-scale, diverse cohorts to ensure their generalisability. Overcoming these challenges is critical to fully realising the potential benefits of these technologies in cardiovascular healthcare.

## Figures and Tables

**Table 1:** Comparing step counting accuracy of 6 activity monitors between healthy individuals and heart failure (HF) patients. Mean absolute percentage error (MAPE) is compared against the criterion device Actigraph wGT3X-BT. Study and data from [24].

Activity Monitor	Release Year	Mean Daily Steps (HF)	MAPE (HF)	Mean Daily Steps (Healthy)	MAPE (Healthy)
Withings Go	2016	4516	18%	Not Reported	Not Reported
Omron HJ-322U	2014	4297	12%	8480	8%
SmartLab Walk+	2014	4299	13%	8573	8%
Garmin Vivofit 1	2014	5921	18%	8562	10%
Garmin Vivofit 3	2016	5671	13%	8393	7%
Fitbit Charge 2	2016	6796	46%	10876	12%

**Table 2:** Summary of requirements applicable for an elderly care indoor localisation system. Table reprinted with permission from [14].

Criterion	Description	Value
Accuracy	2d position compared to reference	0.5–1 m
Installation complexity	Time to install system in a flat	<1 h
User acceptance	Quantitative measure of invasiveness	Noninvasive
Coverage	Area of typical flat	90 m^2^
Update rate	Sampling interval of system	0.5 s
Operating time	Battery life	Not assessed
Availability	The time a system is active and responsive	>90%

## Data Availability

No new data were created or analyzed in this study. Data sharing is not applicable to this article.
